# Galectin-8 as an immunosuppressor in experimental autoimmune encephalomyelitis and a target of human early prognostic antibodies in multiple sclerosis

**DOI:** 10.1371/journal.pone.0177472

**Published:** 2017-06-26

**Authors:** Evelyn Pardo, Claudia Cárcamo, Reinaldo Uribe-San Martín, Ethel Ciampi, Fabián Segovia-Miranda, Cristobal Curkovic-Peña, Fabián Montecino, Christopher Holmes, Juan Enrique Tichauer, Eric Acuña, Francisco Osorio-Barrios, Marjorie Castro, Priscilla Cortes, Claudia Oyanadel, David M. Valenzuela, Rodrigo Pacheco, Rodrigo Naves, Andrea Soza, Alfonso González

**Affiliations:** 1 Center for Aging and Regeneration (CARE), Facultad de Ciencias Biológicas, Pontificia Universidad Católica de Chile, Santiago, Chile; 2 Anatomy and Developmental Biology Program, Institute of Biomedical Sciences, Geroscience Center for Brain Health and Metabolism, University of Chile, Santiago, Chile; 3 Departamento de Neurología, Facultad de Medicina, Pontificia Universidad Católica de Chile, Santiago, Chile; 4 Instituto de Ciencias Biomédicas, Facultad de Medicina, Universidad de Chile, Santiago, Chile; 5 Fundación Ciencia & Vida, Santiago, Chile; 6 Facultad de Ciencias de la Salud, Universidad San Sebastián, Santiago, Chile; 7 Facultad de Ciencia, Universidad San Sebastián, Santiago, Chile; 8 Regeneron Pharmaceuticals, New York, New York, United States of America; 9 Facultad de Ciencias Biológicas, Departamento de Ciencias Biológicas, Universidad Andres Bello, Santiago, Chile; 10 Facultad de Medicina, Universidad San Sebastián, Santiago, Chile; Washington University, UNITED STATES

## Abstract

Galectin-8 (Gal-8) is a member of a glycan-binding protein family that regulates the immune system, among other functions, and is a target of antibodies in autoimmune disorders. However, its role in multiple sclerosis (MS), an autoimmune inflammatory disease of the central nervous system (CNS), remains unknown. We study the consequences of Gal-8 silencing on lymphocyte subpopulations and the development of experimental autoimmune encephalitis (EAE), to then assess the presence and clinical meaning of anti-Gal-8 antibodies in MS patients. *Lgals8*/Lac-Z knock-in mice lacking Gal-8 expression have higher polarization toward Th17 cells accompanied with decreased CCR6^+^ and higher CXCR3^+^ regulatory T cells (Tregs) frequency. These conditions result in exacerbated MOG_35-55_ peptide-induced EAE. Gal-8 eliminates activated Th17 but not Th1 cells by apoptosis and ameliorates EAE in C57BL/6 wild-type mice. β-gal histochemistry reflecting the activity of the Gal-8 promoter revealed Gal-8 expression in a wide range of CNS regions, including high expression in the choroid-plexus. Accordingly, we detected Gal-8 in human cerebrospinal fluid, suggesting a role in the CNS immune-surveillance circuit. In addition, we show that MS patients generate function-blocking anti-Gal-8 antibodies with pathogenic potential. Such antibodies block cell adhesion and Gal-8-induced Th17 apoptosis. Furthermore, circulating anti-Gal-8 antibodies associate with relapsing-remitting MS (RRMS), and not with progressive MS phenotypes, predicting clinical disability at diagnosis within the first year of follow-up. Our results reveal that Gal-8 has an immunosuppressive protective role against autoimmune CNS inflammation, modulating the balance of Th17 and Th1 polarization and their respective Tregs. Such a role can be counteracted during RRMS by anti-Gal-8 antibodies, worsening disease prognosis. Even though anti-Gal-8 antibodies are not specific for MS, our results suggest that they could be a potential early severity biomarker in RRMS.

## Introduction

Multiple sclerosis (MS) is an autoimmune inflammatory disease of the central nervous system (CNS) that damages myelin and axons in the brain and spinal cord [1, 2]. Most (80%) of patients are diagnosed with the relapsing-remitting form of MS (RRMS) and 60% of them evolve towards secondary progressive MS (SPMS) [1]. A subset of patients (20%) exhibits primary progressive MS (PPMS) displaying worse evolution than RRMS patients from the beginning [1]. Such wide variations in the phenotype and aggressiveness of the disease are also reflected in the characteristic heterogeneity in presentation, evolution and treatment responses of RRMS [1, 3, 4]. Although the pathogenic mechanisms remain little understood, during RRMS an autoimmune-driven inflammation of variable intensity and dynamics predominates, underlying the severity of clinical evolution [1]. Observations in RRMS patients, as well as in the MS preclinical mouse model of experimental autoimmune encephalomyelitis (EAE), suggest that disease pathogenesis and resolution depends on a fine balance between the autoimmune inflammation mediated by effector Th1 and Th17 lymphocytes [3, 5] and the immune tolerance promoted by suppressive regulatory T cells (Tregs) [6, 7]. Subpopulations of Tregs expressing either CXCR3 or CCR6 chemokine receptors, respectively, suppress Th1 or Th17-mediated inflammation [8–11], and are recruited to the CNS during neuroinflammatory processes [12, 13], but their role in MS and EAE remains unclear [6]. An additional enigmatic aspect regards the pathogenic role of antibodies, which remains contentious and has mainly been explored focusing on neuronal and myelin elements as antigenic targets [3, 14]. Therefore, modulators of Th1, Th17 and Treg cell homeostasis and their eventual neutralization by function-blocking antibodies entail great interest. Their study might not only reveal new determinants of worse disease evolution but also suggest new prognostic and/or therapeutic approaches.

Galectins are a family of glycan-binding proteins that has emerged as strong modulators of adaptive and innate immunity [15]. These lectins are secreted by an unconventional mechanism and have the potential to modify the function of a variety of cell surface glycoproteins, including signaling receptors and integrins involved in immune-related cellular processes [16–18]. The conserved carbohydrate-recognition domains (CRDs) of galectins recognize β-galactosides, displaying variations that result in unique fine specificities for more complex galactose-containing oligosaccharides and specific downstream effects [19–22]. In this way, different galectins can play redundant or complementary roles in autoimmune diseases by modulating the homeostasis of B and T cells [15], including Th1, Th17 cells and Tregs [15]. Among the 15 members of the galectin (Gal) family, only Gal-1, Gal-3, and Gal-9 have been systematically studied in EAE [20, 23–26].

Gal-8 is a tandem-repeat galectin that, unlike other galectins, possesses unique high affinity for α-2,3-sialylated glycans in its N-terminal carbohydrate recognition domain (CRD), which is linked by a short peptide to a C-terminal CRD bearing another glycan specificity [27, 28]. Gal-8 has been proposed to play immunosuppressive roles inducing apoptosis of activated T cells [29], including Th17 cells [30], and promoting differentiation of immunosupressive Tregs [30, 31]. Treatment with Gal-8 ameliorates Th1 and Th17-mediated ocular pathology in experimental autoimmune uveitis (EAU) [30]. Altered Gal-8 functions have been found in rheumatic, autoimmune and inflammatory human disorders [32, 33]. A single aminoacid polymorphism (F19Y) with functional implications on cancer cell growth [34] and glycan interactions [35], strongly associates with rheumatoid arthritis [36]. Interestingly, neutralizing anti-Gal-8 autoantibodies that block Gal-8 interactions with glycans on β1-integrins and LFA-1 [37, 38], as well as Gal-8-induced apoptosis of T cells [29], are frequently generated in systemic lupus erythematosus (SLE), the prototypic autoimmune disease, and also in rheumatoid arthritis and probably other inflammatory disorders [39]. However, the role of Gal-8 and its neutralizing antibodies have not yet been explored in MS.

Here we study: 1) How the absence of Gal-8 function in a Gal-8 KO mice impinges upon lymphocyte subpopulations and EAE severity; 2) The effect of Gal-8 treatment on EAE of wild type mice; 3) The presence and clinical meaning of autoantibodies against Gal-8 in patients with MS. Our results indicate a protective immunosuppressive role of Gal-8 against CNS autoimmunity involving apoptotic elimination of activated Th17 cells and opposite dysbalances of CXCR3^+^ and CCR6^+^ Treg frequencies. Strikingly, MS patients generate neutralizing anti-Gal-8 antibodies that can block the Gal-8 immunosuppressive function and impact upon RRMS prognostic. Patients recently diagnosed with RRMS and harboring anti-Gal-8 antibodies in serum, before establishing the treatment, develop a more aggressive disease within just one year of evolution, as shown by monitoring the Expanded Disability Status Scale (EDSS). Overall, the evidence suggests that Gal-8 plays an immunosuppressive function that can be counteracted by anti-Gal-8 antibodies in RRMS leading to worse evolution. Although Gal-8 function-blocking antibodies are not specific to MS, as they can be found in other autoimmune diseases [39], their presence in context of RRMS might be useful as an early biomarker of worse evolution.

## Material and methods

### Study approval

The Scientific Ethics Committee CEC-MED-UC and the Science Ethics Committee for Animal and Environmental Care of the Pontificia Universidad Católica de Chile (PUC) approved the informed consent voluntarily given by MS patients and all animal procedures, respectively.

### Patients and clinical variables

MS patients were monitored by trained neurologists in the Center of Multiple Sclerosis PUC, between 2011 and 2013, during standard clinical visits, by collecting demographic data, relevant clinical history, neurologic assessments, including the Expanded Disability Status Scale (EDSS), relapse symptoms and Magnetic Resonance Imaging (MRI) using 1.5 Tesla MRI with 5 mm thick slices and 2.5 mm separation between each slice. Clinically relevant EDSS worsening implied changes from 0 to 1.5 or increments of 1 point within the range of 1 to 5.5 scores. Blood samples were obtained from RRMS patients before starting treatment with any disease-modifying drugs (DMD). Patients with progressive forms were not receiving any DMD or immunosuppressant drugs at least 6 months before the blood samples. No steroid treatment was used in RR, SP or PP patients for at least 4 weeks prior to the blood sample. Patients were followed up for an average of one year, controlled every 3 to 6 months, or more frequently (e.g. during relapses), as needed. All patients accepted and signed the informed consent approved by the Scientific Ethics Committee CEC-MED-UC.

### Antibodies and reagents

To determine expression levels of key surface and intracellular molecules associated with different T-cell phenotypes, splenocytes were immunostained with different combinations of the following fluorochrome-conjugated monoclonal antibodies (mAbs) at room temperature for 30 min: allophycocyanin (Apc)-conjugated anti-FoxP3 (clone FJK-16S) and R-Phycoerythrin (PE)-conjugated anti-RORγt (clone BSD), both from eBioscience (San Diego, CA). Otherwise, PerCP/Cyanine dye 5.5 (PerCP/Cy5.5)-conjugated anti-CCR6 (clone 29-2L17), PE/Cyanine dye 7 (PE/Cy7)-conjugated anti-CD4 (clone GK1.5), Brilliant-Violet 421^TM^ (Bv421)-conjugated anti-CXCR3 (Clone CXCR3-173), PE-conjugated anti-CD44 (clone IM7), APC-conjugated anti-CD25 (clone PC61) and APC/Cyanine dye 7 (APC/Cy7)-conjugated anti-CD62L (clone MEL14) were purchased from Biolegend (San Diego, CA). PerCP-conjugated anti-CD4 (clone RM4-5), PECy7-conjugated IFN-γ (clone XMG1.2) were purchased from eBioscience. Alexa 647-conjugated IL-17A (clone TC11-18H10.1), PE-conjugated anti-IFN-γ and Alexa 488–conjugated anti-IL-17 were purchased from BioLegend. Alexa 488-conjugated anti-CD8a (clone 53–6.7), alexa 647-conjugated anti-CD19 (clone 1D3), PE-conjugated anti-CD11c (clone HL3), PE-conjugated anti-IL4 (11B11), Alexa 647-conjugated anti-IL17A (clone TC11-18H10), PE/Cy7-conjugated anti-IFN-γ (cloneXMG1.2) were purchased from BD Biosciences-Pharmingen. For T cell activation, anti-mouse CD28 (clone 37.51) and anti-mouse CD3 (clone 145-2C11) were purchased from BD Bioscience.

Ionomicina, brefeldin A and PMA (Phorbol Myristate Acetate) were purchased from Sigma-Aldrich. GST-Gal-8 expressed in bacteria was isolated by affinity chromatography and Gal-8 was released by thrombin treatment as described [37].

### Animals

Mice were mantained under conditions of strict confinement, which included automatic control of temperature (21°C) and photoperiod (12 h light / 12 h dark). *Lgals8*/Lac-Z knock-in (here called Gal-8 KO or *Lgals8*^-/-^) mice were generated from *C57BL/6NTac* mice engineered in Regeneron Pharmaceuticals Inc., New York, using Velocigene technology for replacing the entire coding region of the mouse *Lgals8* gene (18,427 bp) with LacZ lox-Ub1-EM7-Neo-lox Cassette containing the LacZ gene that encodes β-galactosidase [40]. Details of the *Lgals8* KO mice and PCR genotyping assay, including the predicted PCR products and the primers, are available at the Velocigene website (www.velocigene.com/komp/detail/14305). IL-17A-GFP reporter mice in the C57BL/6J background, which express EGFP under the control of the IL-17A promoter, were purchased from Jackson Laboratories (Bar Harbor, ME).

### EAE induction, scoring and treatment

EAE was induced in 8-12-week-old *Lgals8*^-/-^ and *Lgals8*^+/+^ mice by immunization of an emulsion containing 150 μg of MOG_35-55_ peptide (MOGp) (Biosynth International) and 500 μg of Mycobacterium tuberculosis extract (Difco Lab.) in incomplete Freund’s adjuvant oil. In addition, mice received 200 ng of pertussis toxin (List Biological Laboratories) on day 0 and 2 post-immunization (p.i.). Clinical progression of EAE symptoms were monitored daily using the following scale: 0, no clinical signs; 1, loss of tail tone; 2, flaccid tail; 3, incomplete paralysis of one or two hind legs; 4, complete hind limb paralysis; 5, moribund (animals that do not move, do not consume water or food, that loss weight greater than 20% or have respiratory problems, were euthanized); 6, death. In some experiments, EAE was induced in wild-type C57BL/6J mice and simultaneously treated (i.p.) with either 100 μg recombinant Gal-8 or PBS (control group) during 20 consecutive days. Topical diclofenac (gel 1%) was used to alleviate animal suffering. The use of local analgesia rather than systemic analgesia was favored to avoid unwanted influences on immunological and inflammatory parameters under study. The animals were euthanized by cervical dislocation after CO_2_ sedation.

### CNS ß-gal histochemistry and histological analysis

Brains were fixed by perfusion in 4% paraformaldehyde (PFA) in PB buffer (0.1 M phosphate buffer pH 7.4) and processed for β-gal histochemistry as described [41, 42]. Cross-sections of spinal cords were obtained from mice at 20 days after EAE induction and immersion-fixed in neutral buffered formalin solution. Inflammatory infiltrates and demyelination were histochemically analyzed by hematoxylin and eosin (H&E) and luxol fast blue staining, respectively.

### Subpopulation analysis of splenocytes

Splenocytes were isolated as described [43, 44] from 8-12-week-old female mice and analyzed by FACS. For polyclonal T cell activation, splenocytes were grown in the presence of 1 μg/ml of anti-CD3 with or without anti-CD28 antibodies for 72 h. For Gal-8 effects on antigen-specific T cell activation, splenocytes were isolated after 10 days of EAE induction and re-stimulated with 10 μg/ml MOGp in the presence or absence of 100 μg/ml recombinant Gal-8 for 72 h. CD4^+^ T cell subpopulations were analyzed in stimulated cells by incubating with 50 ng/ml PMA, 500 ng/ml ionomycin, and 10 μg/ml brefeldin A for the last 4 h of culture. Cells were stained with Zombie Aqua Fixable Viability kit (Biolegend) in PBS followed by staining for cell-surface markers using flurophore-conjugated monoclonal antibodies against mouse CD4, CD19, CD8, CD11c, CD25, CD44, CD62L, CXCR3 and CCR6. For intracellular staining, cells were washed, fixed, and permeabilized with Cytofix/Cytoperm kit (BD Biosciences-Pharmingen) and incubated simultaneously with fluorophore-conjugated antibodies against IFN-γ, IL-4, IL-17, Foxp3 and RORγt. Samples were acquired with BD FACSVerse or FACSCanto^TM^ II (BD) flow cytometers and data was analyzed with FlowJo V10 software or CellQuest Pro software (BD Biosciences).

### Th17 cell death analysis

To analyze apoptosis of Th17 cells differentiated *in vitro*, naive (CD62L^+^ CD44^-^) CD4^+^ T-cells from IL-17A-GFP reporter or from wild-type mice were purified by cell sorting, resuspended at 0.5x106 cells/ml in IMDM medium supplemented with antibiotics/antimycotics (Gibco) and Gentamycin (Gibco), and incubated (200 μl/well) in 96-well plates preactivated with anti-CD3 (50 μl/well at 2 μg/ml) at 37°C, 90% humidity and 5% CO2. Cells were incubated for 3 days with mouse IL-6 (mI-6; 25 ng/mL), mIL-1ß (20 ng/ml), human TGF-β1 (hTGF-β1; 5 ng/ml), anti-CD28 (2 μg/ml) and blocking antibodies anti-IFNγ, anti-IL-4 and anti-IL-2 (5 μg/ml each). Afterwards, cells were washed, resuspended in fresh medium and incubated for additional 4 days in 96-well plates with mIL-6 (25 ng/ml) and hTGF-β1 (5 ng/ml). All cytokines (Biolegend) used were carrier free. Differentiated Th17 cells were purified by cell sorting based on IL-17A expression (GFP^+^), resuspended in supplemented IMDM and incubated (1x10^5^ cells/well) in 96-well plates pre-activated with anti-CD3 and anti-CD28 (50 μl/well at 2 μg/ml each) in the presence or absence of Gal-8 (20 μg/ml) for 72 h. The extent of apoptosis/necrosis was determined with a commercial kit (Pacific Blue TM Annexin V Apoptopsis Detection Kit with 7-AAD; 640926, Biolegend) analyzing death cells by FACS, in the CD4^+^ GFP^+^ gated population corresponding to ≥70% of total cells, as observed in FSC versus SSC analysis. Gal-8-induced cell death of Th17 lymphocytes was analyzed with a similar protocol, except that naive CD4^+^ T-cells were isolated from WT C57BL/6 mice, differentiated Th17 cells were purified by using a commercial kit (from Biolegend) based on the surface expression of IL-17. Cell death (Annexin V versus 7-AAD) was analyzed in the CD4^+^ gated population, which corresponded to ≥90% of total cells, accordingly to FSC versus SSC analysis.

### Detection of anti-Gal-8 autoantibodies

Western blot analysis was performed using recombinant Gal-8 to screen sera of patients with MS for anti-Gal-8 antibodies, as previously described in patients with SLE or AR [39]. The same positive and negative controls were included in each western blot and densitometric analysis of the bands was performed by ImageJ. Bands that duplicate the intensity of the negative control in at least two independent experiments performed in triplicate and blinded with respect to the sample were considered positive for anti-Gal-8 reactivity.

### Detection of Gal-8 in human cerebro spinal fluid (CSF)

CSF collected for diagnostic purposes from individuals studied for diplopia, vertiginous syndrome (C7), cephalea, febrile syndrome and meningitis was analyzed for the presence of Gal-8 by immunoblot, using a rabbit anti-Gal-8 polyclonal antibody produced in our laboratory [45]. CSF (1 ml) was centrifuged for 10 min at 1,000 g to eliminate debris and the supernatant was incubated with 50 μl of α-lactose-agarose beads for 3 h at 4°C in presence of anti-proteases (2 μg/ml leupeptin, 2 μg/ml pepstatin and 2 mM PMSF). Lectins bound to α-lactose-agarose beads were pulled down at 1,000 rpm for 3 min, the beads were washed three times with PBS and suspended in 40 μl of electrophoresis loading buffer. The samples were heated for 5 min at 100°C and loaded in 10% SDS-PAGE, transferred to nitrocellulose membranes and analyzed with anti-Gal-8 antibodies.

### Isolation of human peripheral blood mononuclear cells (PBMC)

Ten milliliters of peripheral blood was diluted with 10 ml of PBS, layered carefully over 7.5 ml of Histopaque-1077 (Sigma Chem. Co.) and centrifuged at 400 x g for 30 min at room temperature. Cells in the opaque interface were collected, diluted twice with 50 ml of PBS, centrifuged at 250 x g for 10 min and resuspended in RPMI 1640 medium containing 10% FBS.

### Cell adhesion assay

Cover slips were coated overnight at 4°C with 10 μg/ml of Gal-8 in the absence or presence of anti-Gal-8 autoantibodies, as described [37, 38, 46]. Serum-starved PBMCs isolated from healthy people (200,000 cells/well) were plated on cover slips for 30 min at 37°C. Adherent cells were stained with Hoechst and quantified analyzing at least three random fields on three cover slips.

### Statistical analysis

The presence of anti-Gal-8 autoantibodies in RRMS, PPMS and SPMS patients was analyzed respect to various clinical, radiological and CSF variables using Mann-Whitney U test (with Tukey multiple comparisons) and SPSS Statistics 19.0 software. Yates chi-square corrected test and Odds ratio were calculated with VassarStats. EAE disease scores were analyzed with Mann–Whitney U test or one-way ANOVA Kruskal Wallis with Dunn post-test using Prism 5 software (GraphPad Software). Other analyses were made with two tailed non-paired Student’s *t*-test as indicated in figure legends.

## Results

### Endogenous Gal-8 protects against EAE

**Fig 1:**
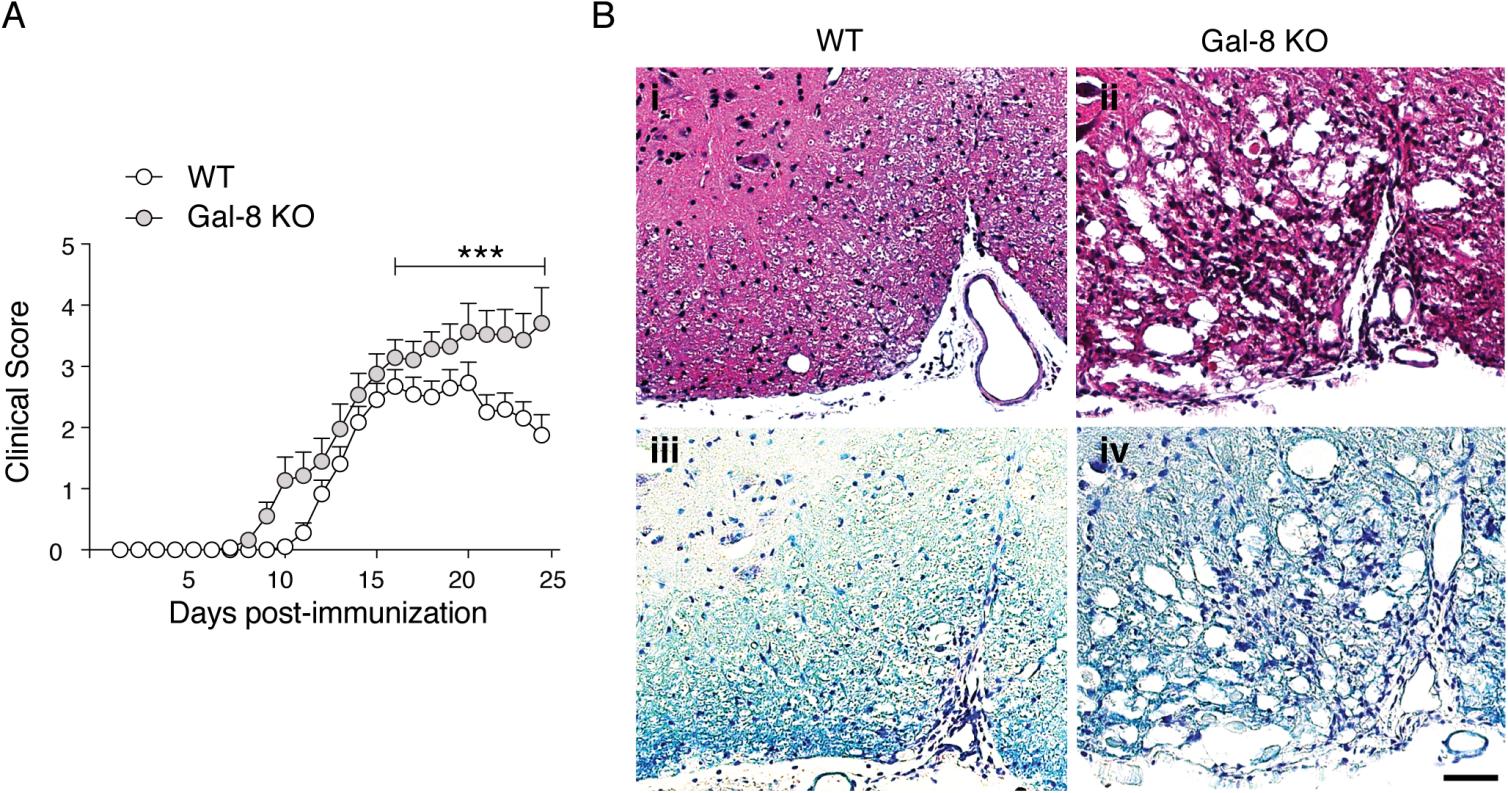
Lack of endogenous Gal-8 expression exacerbates EAE. *Lgals8*^+/+^ (WT) and *Lgals8*^-/-^ (Gal-8 KO) immunized with MOGp. (A) Clinical scores monitored daily for 25 days show an exacerbated EAE in Gal-8 KO mice (***p<0.001; Mann Whitney; day by day clinical Score comparisons; n = 20 WT; n = 22 Gal-8 KO). (B) Spinal cord histopathological analysis after 10 days of immunization show enhanced immune cell infiltration and demyelination in Gal-8 KO mice, shown by H&E staining (I and II) and luxol fast blue staining (III and IV). Scale bar = 50 μm.

To assess the role of Gal-8 in autoimmune CNS pathogenesis we first study how *Lgals8* disruption affects EAE severity. We evaluated the development, progression and CNS inflammation in *Lgals8*^-/-^ and *Lgals8*^+/+^ littermates immunized with MOG_35-55_ peptide (MOGp). The two mouse groups had similar EAE incidence (85.2% *Lgals8*^+/+^ littermates and 86.2% *Lgals8*^-/-^). However, *Lgals8*^-/-^ mice displayed a faster disease onset and developed a more severe disease during the chronic phase (Fig 1A and Table 1). Spinal cord histological analysis showed enhanced inflammation (Fig 1B, i and ii) and demyelination (Fig 1B, ii and iv) in the affected areas of *Lgals8*^-/-^ mice. Such EAE exacerbation under Gal-8 deficiency suggests a protective role of endogenous Gal-8 against CNS autoimmunity.

**Table 1:** Clinical parameters of EAE progression.

Group of mice	n	Incidence (%)	Day of Onset (Mean ± SD)	Maximum Score (Mean ± SD)	Time to Peak (Mean ± SD)	Accumulative Score (Mean ± SD)
***Lgals8***^***+/+***^	20	85.2	13.5 ± 2.8	3.4 ± 0.7	15.9 ± 3.4	28.2 ± 11.9
***Lgals8***^***-/-***^	22	86.2	12.5 ± 2.6	3.6 ± 0.6	15.5 ± 3.2	40.7 ± 23.2*

*P < 0.05 comparing accumulative scores between WT and Gal-8 KO mice.

### Gal-8 silencing leads to increased Th17 cells and Th1-like Tregs

**Fig 2:**
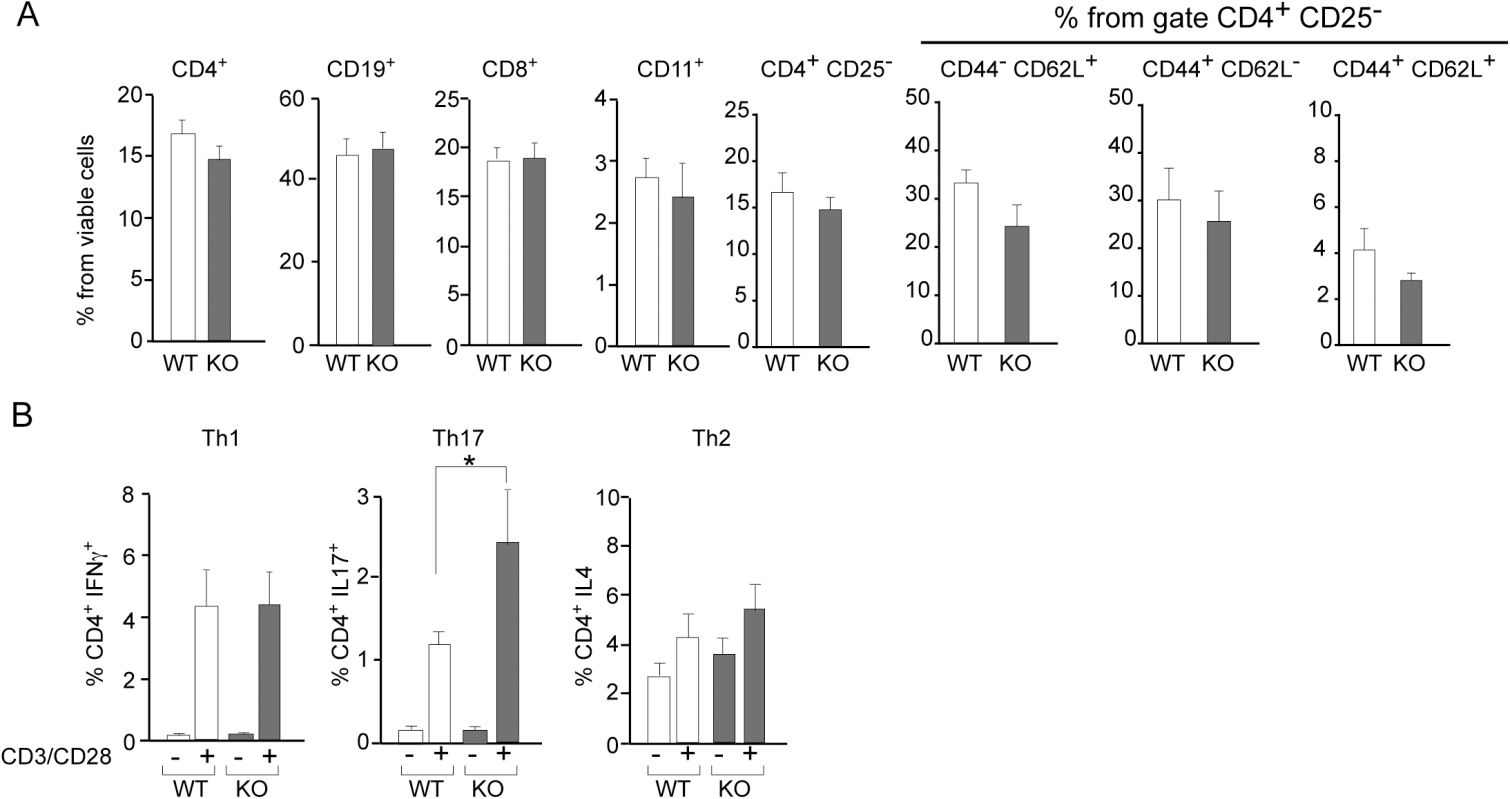
Gal-8 deficit favors selective Th17 cell differentiation upon polyclonal activation. Splenocytes isolated from *Lgals8*^+/+^ (WT) and *Lgals8*^-/-^ (KO) mice were analyzed by FACS: (A) Dendritic cells (CD11c^+^), B cells (CD19^+^), CD8^+^ T cells and different CD4^+^ T cells subsets, naïve (CD44^-^CD62L^+^), effector (CD44^+^CD62L^+^), memory (CD44^+^CD62L^-^) and total cells analyzed in the subset of viable CD4^+^ CD25^-^ T-cells show no differences between WT and KO mice. Graphics of frequency +/-SD (n = 5). (B) T cell activation by 72 h incubation with anti-CD3 (anti-CD3) and anti-CD28 (anti-CD28) antibodies show Th17 increased frequency in KO mice while Th1 and Th2 cells are similar in WT and KO mice. Graph shows frequency +/-SD (*p<0.05; ANOVA; n = 4).

To understand how the lack of Gal-8 predisposes the immune system to a more pronounced CNS autoimmunity we compared lymphocyte subpopulations and the responses of spleen cells to *ex vivo* anti-CD3/anti-CD28 activation and to re-stimulation with MOGp. Splenocytes from *Lgals8*^-/-^ and *Lgals8*^+/+^ littermates showed similar frequencies of B and T (CD4^+^ and CD8^+^) lymphocytes, dendritic cells, and CD4^+^ T-cells subsets, including naive (CD4^+^CD25^-^ or CD4^+^ CD62L^+^ CD44^-^), effector (CD4^+^ CD62L^-^ CD44^+^) and memory (CD4^+^ CD62L^+^ CD44^+^) T-cells (Fig 2A). However, *Lgals8*^-/-^ splenocytes displayed higher polarization towards Th17 (CD4^+^ IL17^+^) after anti-CD3/anti-CD28 activation (Fig 2B).

**Fig 3:**
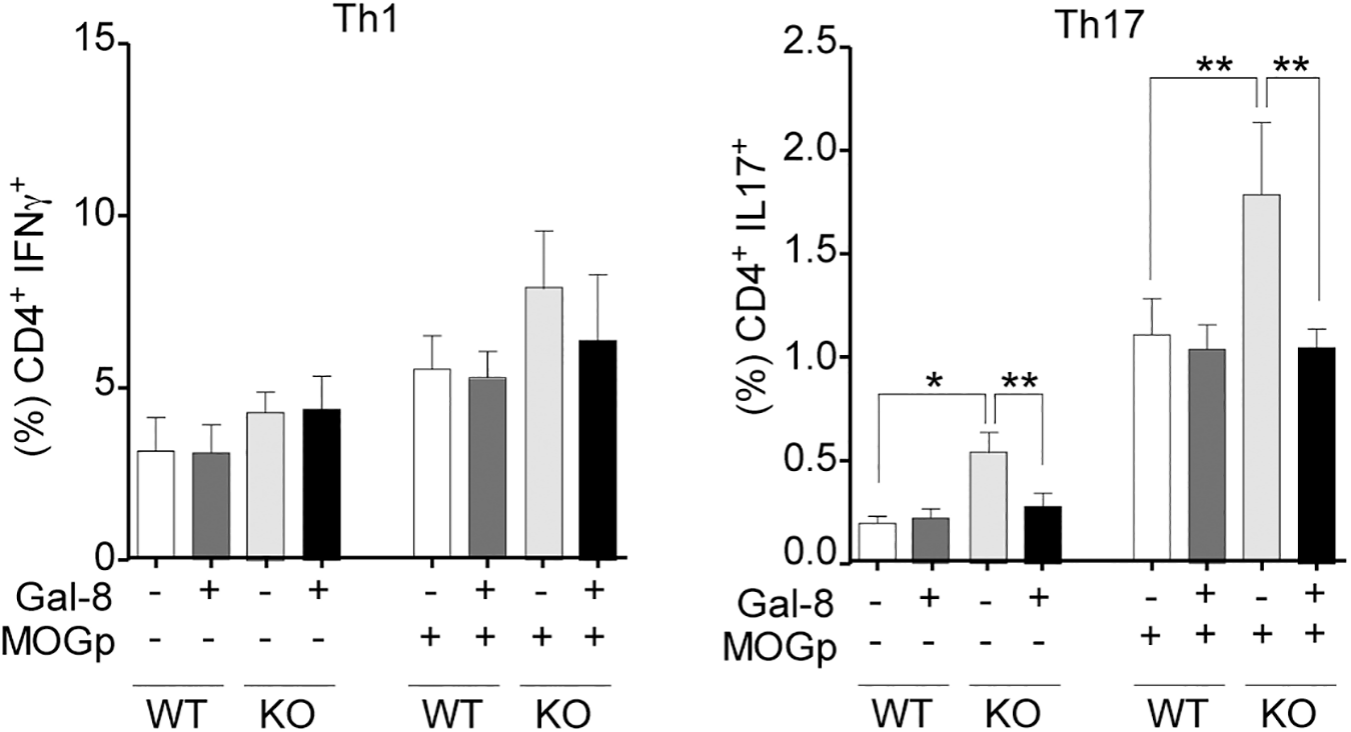
Gal-8 deficit favors Th17 polarization during MOGp-induced EAE and *ex-vivo* re-stimulation. Th17 and Th1 subpopulations in splenocytes from *Lgals8*^-/-^ (KO) and *Lgals8*^+/+^ (WT) mice obtained after 10 days of EAE induction were analyzed either immediately or after 72 h of *ex vivo* MOGp re-stimulation, in the absence or presence of Gal-8. Gal-8 KO mice show higher frequency of Th17 cells both at steady state and after MOGp re-stimulation. Incubation with Gal-8 reduced Th17 cells only in Gal-8 KO. Graph shows frequency +/-SD (*p<0.05; ANOVA; n = 4).

We then compared the systemic expansion of Th1 and Th17 cells during EAE induction by analyzing the splenocytes from *Lgals8*^-/-^ and *Lgals8*^+/+^ mice after 10 days post-immunization and *ex vivo* re-stimulation with MOGp. We did not detect meaningful differences in the frequency of Th1 cells between *Lgals8*^-/-^ and *Lgals8*^+/+^ mice (Fig 3). Th1 cells also remained unaffected under *ex vivo* Gal-8 incubation (Fig 3). In contrast, splenocytes from *Lgals8*^-/-^ mice unstimulated or re-stimulated with MOGp *ex vivo* showed an increased frequency of Th17 cells compared with *Lgals8*^+/+^ littermates (Fig 3). Incubation with exogenous Gal-8 reduced the frequency of Th17 cells in *Lgals8*^-/-^ splenocytes to the levels of control mice (Fig 3), thus contrasting with the lack of response seen in *Lgals8*^+/+^ splenocytes. Therefore, in the absence of endogenous Gal-8 expression there is increased Th17 polarization, while in the presence of endogenous Gal-8, the Th17 cells activated by MOGp immunization and likely involved in EAE seem to be eliminated.

**Fig 4:**
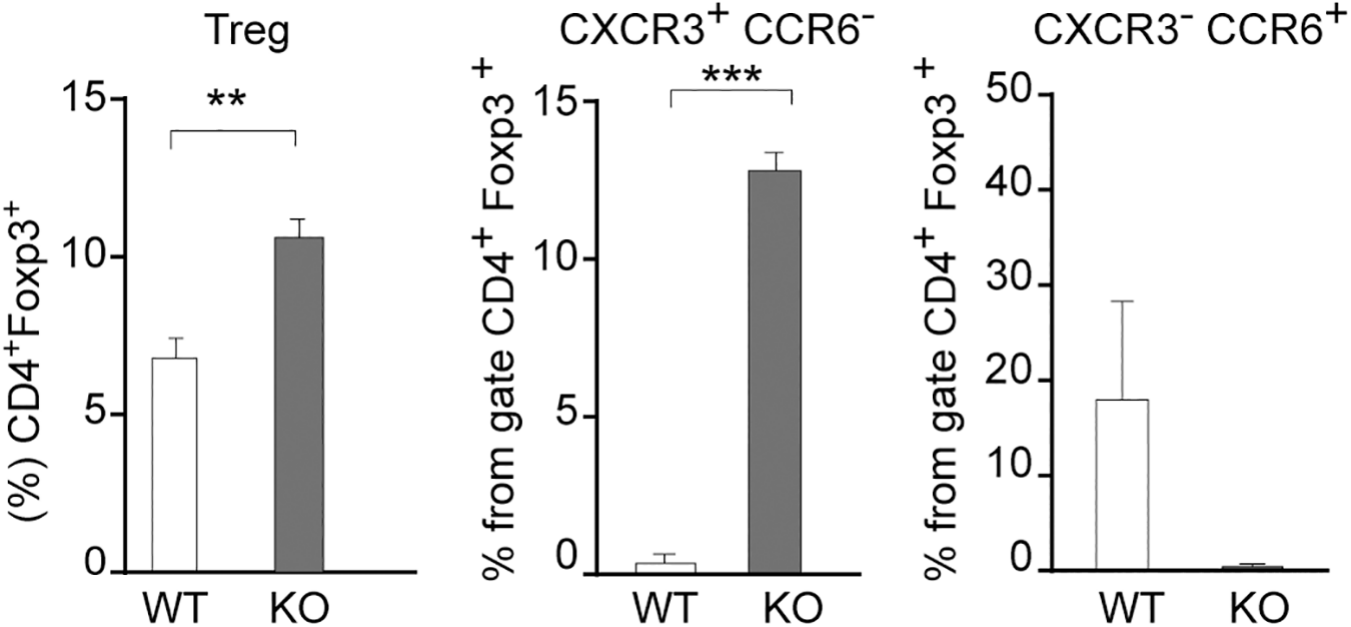
Galectin-8 deficit increases the frequency of total Tregs and CXCR3^+^ Tregs. Splenocytes isolated from *Lgals8*^+/+^ (WT) and *Lgals8*^-/-^ (KO) mice were analyzed at steady state for total Tregs (Foxp3^+^), CXCR3^+^ and CCR6^+^ frequency in the Treg (Foxp3^+^ CD4^+^) population. Graphs of frequency +/-SEM show increased total Tregs (Foxp3^+^) and CXCR3^+^Tregs in KO mice (*p<0.05; **p<0.01; p***<0.001; Student’s *t*-test; n = 4).

As Tregs have been shown to control Th17 and Th1-mediated tolerance and inflammatory responses in MS and EAE [6], we analyzed total Tregs, as well as Tregs subpopulations that suppress responses mediated by either Th1 (CXCR3^+^ CCR6^-^) [9, 10] or Th17 (CXCR3^-^ CCR6^+^) Tregs [11] lymphocytes. Unexpectedly, we found an increased frequency of total Tregs (Foxp3^+^) in *Lgals8*^-/-^ mice splenocytes compared to control mice (Fig 4). We also found a highly increased frequency of CXCR3^+^ Tregs, whereas CCR6^+^ Tregs tended to decrease in *Lgals8*^-/-^ compared with control *Lgals8*^+/+^ mice (Fig 4). Therefore, the absence of Gal-8 leads to an increase of Th17 polarization, as well as higher generation of CXCR3^+^ Tregs and lower frequency of CCR6^+^ Tregs, which respectively would impact upon Th1 and Th17 functions [8].

### Gal-8 ameliorates EAE and induces apoptosis of activated Th17 cells

**Fig 5:**
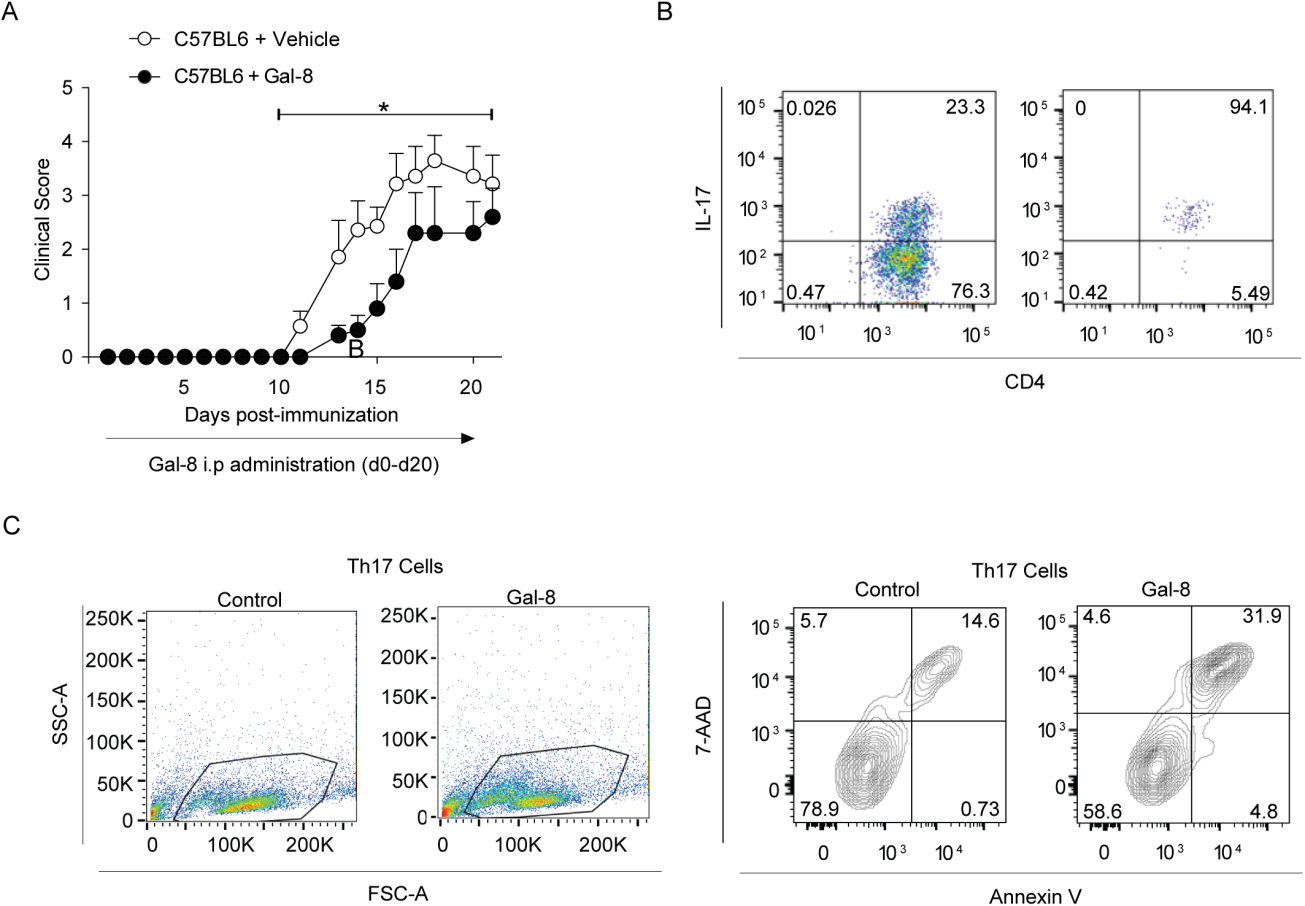
Gal-8 ameliorates EAE and induces Th17 cell death *in vitro*. (A) Gal-8 treatment ameliorates MOGp-induced EAE in C57BL/6 mice. The mice were injected daily by intraperitoneal injection of either PBS (Control) or Gal-8 100 μg/ml. Gal-8-treated animals tend to start the disease later and show lower EAE scores during the acute and chronic phases of the disease (*p<0.05; Control, n = 7; Gal-8 treated, n = 5). (B) Gal-8-induced cell death in Th17 lymphocytes differentiated and activated *in vitro*. Naive (CD62L^+^ CD44^-^) CD4^+^ T cells were purified by cell sorting and differentiated to a Th17 phenotype. The differentiated Th17 cells were isolated with a commercial kit based on the cell surface expression of IL-17 and activated with anti-CD3/anti-CD28 in the presence or absence of Gal-8 (20 μg/ml) for 72 h. Cell death was determined by cell staining with Annexin V and 7-AAD and analyzed by FACS. Representative dot plots show the frequency of Th17 cells before and after purification (upper panels), the selected gate in the forward scatter versus side scatter analysis (middle panels) and the associated contour-plots show the Annexin V versus 7-AAD analysis (lower panels). Numbers in quadrants indicate the percentage of cells in the respective quadrant.

The key role of endogenous Gal-8 in regulating the functions of effectors and Tregs during EAE development prompted us to evaluate the impact of the exogenous Gal-8 treatment on EAE induction and Th17 survival in wild-type C57BL/6 mice. Daily treatment with Gal-8 starting from disease induction significantly delayed the progression of EAE clinical symptoms (Fig 5A and Table 2). To test the sensitivity of wild-type activated Th17 cells to Gal-8 treatment, we differentiated Th17 cells *in vitro* and activated them with anti-CD3/anti-CD28. Annexin V/7-AAD staining showed that Gal-8 induced apoptosis of these Th17 activated cells (Fig 5B). These results indicate that exogenous Gal-8 exert immune-suppressive action against EAE induction involving apoptotic elimination of activated Th17 cells.

**Table 2:** Clinical parameters of EAE disease progression (day 0–20).

Group of mice	n	Day of Onset (Mean ± SD)	Maximum Score (Mean ± SD)	Time to Peak (Mean ± SD)	Accumulative Score (Mean ± SD)
**Vehicle**	7	12.7 **±** 1.7	4.1 **±** 0.8	16.4 **±** 2.4	20.8 **±** 8.0
**Gal-8 treatment**	5	13.6 **±** 0.9	2.8 **±** 1.6	17.8 **±** 1.4	10.1 **±** 7.0*

*P < 0.05 comparing accumulative scores of mice injected with either vehicle or Gal-8.

### Gal-8 expression in the brain

**Fig 6:**
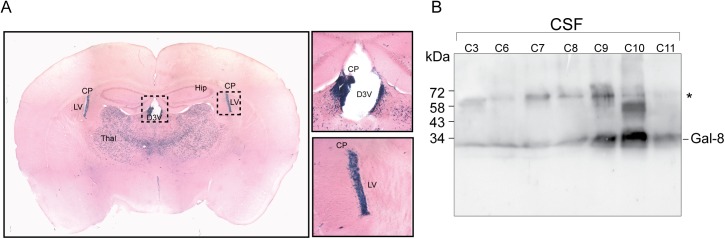
Gal-8 expression in mouse brain and presence in human CSF. (A) Histochemistry of β-gal staining reveals Gal-8 expression in several regions of the mouse brain (S1 Table). Brain slices depict high Gal-8 expression levels in the choroid plexus (CP) of the lateral ventricle (LV) and the dorsal 3rd ventricle (D3V), as well as in the ventrolateral thalamic nucleus. (B) Immunoblot with rabbit anti-Gal-8 antibody show Gal-8 reactivity in the CSF of individuals without MS. Samples C3-11 correspond to non-inflammatory CSF from individuals studied for diplopia (C3), vertiginous syndrome (C7), cephalea (C8 and C11) and febrile syndrome (C9), whereas C6 is an inflammatory CSF from a patient with meningitis. All samples show anti-Gal-8 reactivity, though with variable intensity. *Bands of unknown origin might include Gal-8 dimers or complexes with other proteins, not separable under SDS-PAGE conditions.

Although peripheral events tailoring the immune system contribute to MS and EAE pathology, most of the autoimmune pathogenic condition unfolds inside the CNS [3]. In our knock-in *Lgals8* mice the β-galactosidase (β-gal) cassette reporter gene replaces the entire Gal-8 gene with LacZ, thus offering the possibility to assess the activity of the corresponding promoter by β-gal histochemistry [40–42]. This analysis revealed Gal-8 expression in several brain regions (Fig 6A; S1 Table). Interestingly, the choroid plexus, which generates CSF [47], displayed high expression levels, suggesting that Gal-8 might be secreted into the CSF. To test this possibility we analyzed CSF from patients studied for other pathologies, mainly cephalea, and included one patient with meningitis. We detected Gal-8 in all CSF samples with variable intensity. The highest levels corresponded to a patient studied for cephalea (Fig 6B). These results suggest that Gal-8 produced by the choroid-plexus is a component of the CSF.

### Patients with MS generate function-blocking antibodies against Gal-8

**Fig 7:**
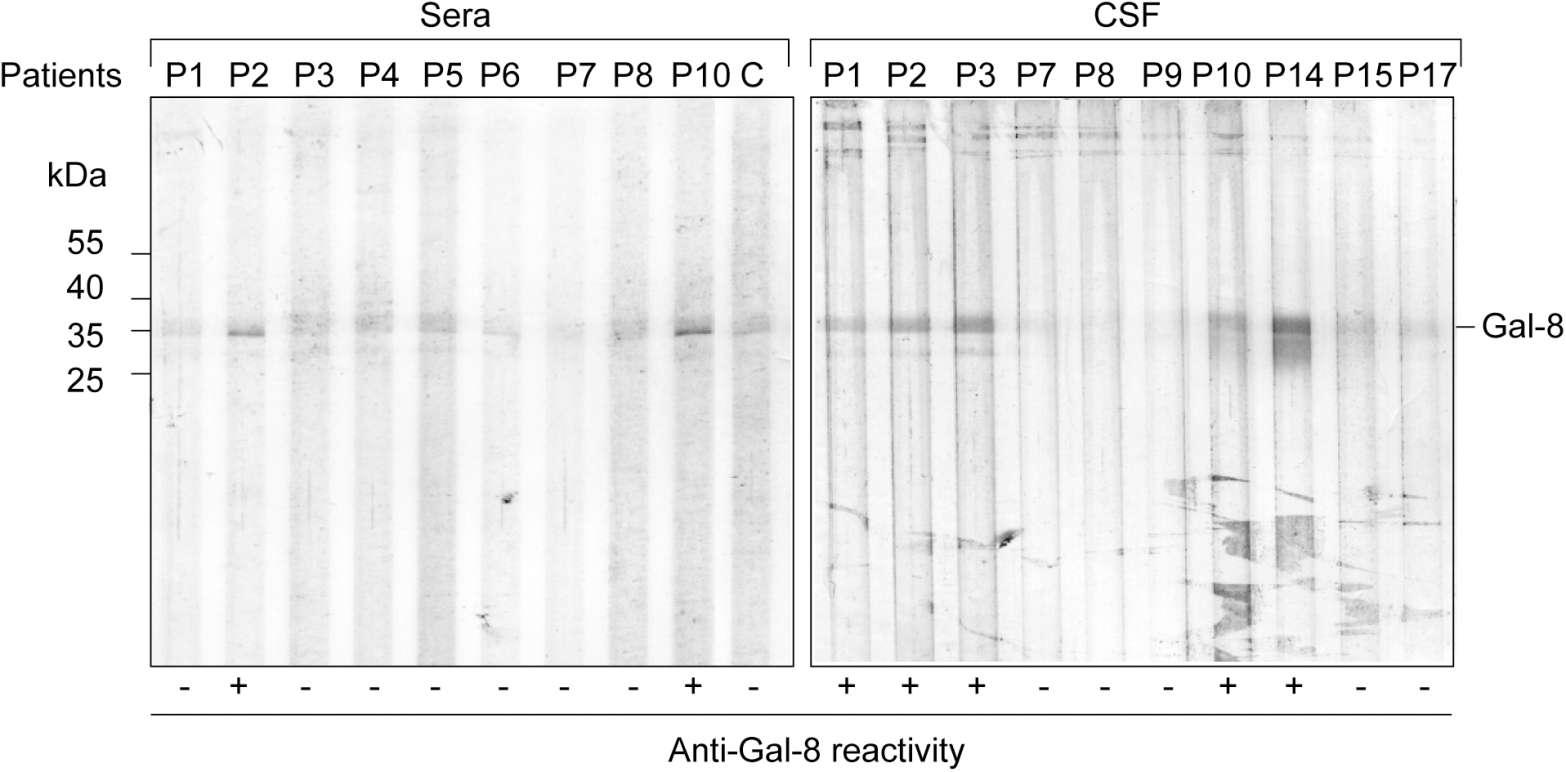
Detection of Gal-8 autoantibodies in sera and CSF from MS patients. Immunoblot of sera and CSF from different MS patients (Pn) against Gal-8 indicating its (-) or (+) anti-Gal-8 reactivity compared with a negative control (C) from a healthy individual. In some patients (e.g. P2 and P10) anti-Gal-8 reactivity was detected in both sera and CSF, while in others (e.g. P3) was only detected in CSF. P14 is shown only in CSF but is also positive in serum (analyzed in other immunoblot), while P15 and P17 are negative both in CSF and serum (not shown). In most patients (e.g. P4-6) only sera could be analyzed. P9 was only analyzed in CSF.

The results showing an immunosuppressive and protective role of Gal-8 against EAE prompted us to assess whether patients with MS generate blocking-function anti-Gal-8 antibodies, as previously reported in LES and AR patients [39, 46]. Using recombinant human Gal-8 [37] and immunoblot analysis [39] we found clear evidence of anti-Gal-8 autoantibodies in a cohort of RRMS patients (Fig 7). We also found evidence of the presence of anti-Gal-8 antibodies in CSF, either coincident or independent of serum reactivity (Fig 7).

**Fig 8:**
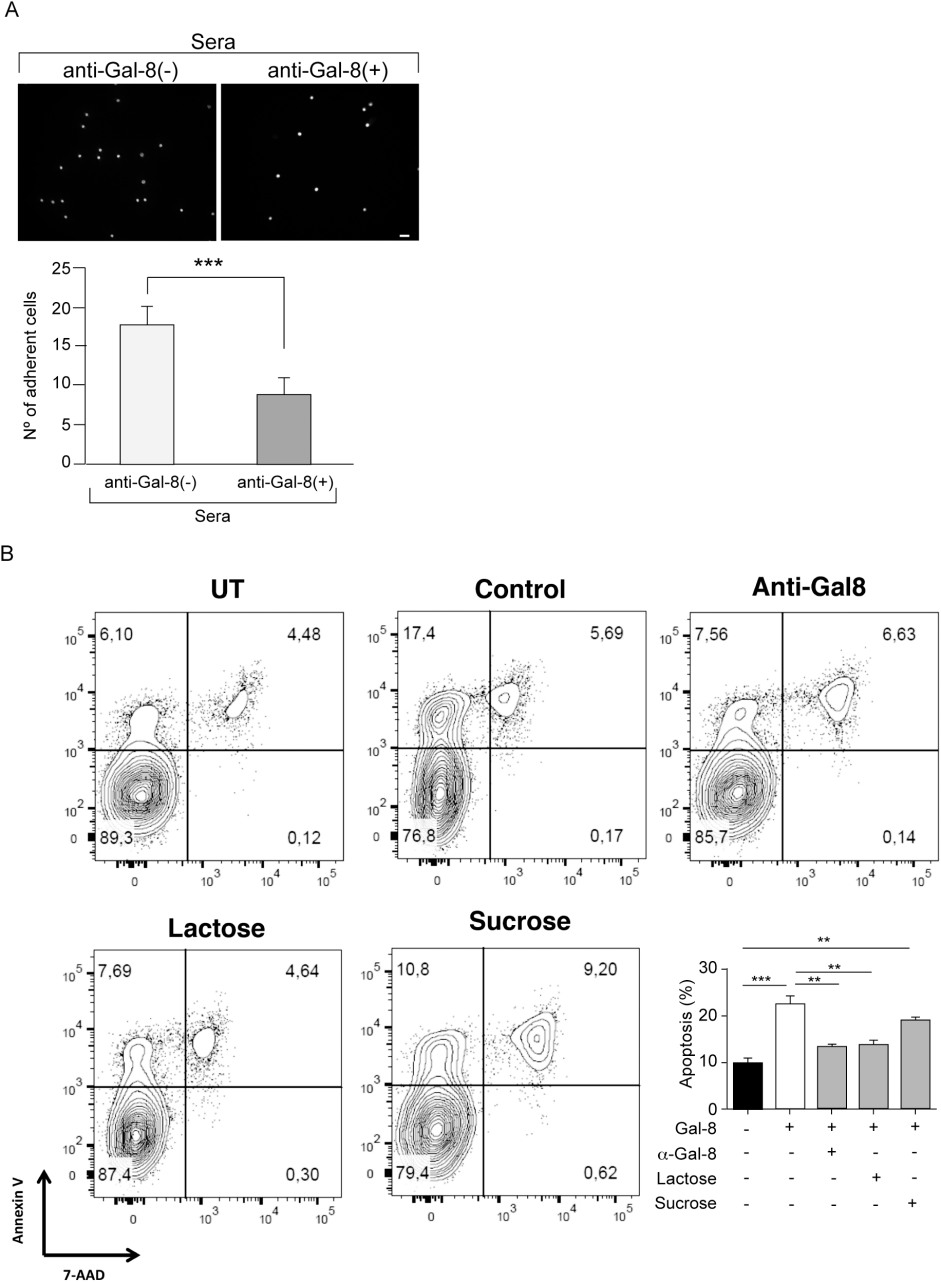
Function-blocking activity of anti-Gal-8 autoantibodies. (A) Anti-Gal-8(+) sera block the adhesion of PBMC to Gal-8-coated coverslips. Graph shows number of adhered cells (Average ± SE of three anti-Gal-8(-) and three anti-Gal-8(+) sera tested in triplicate) (***p<0.001; Student’s *t*-test). (B) Anti-Gal-8 autoantibodies inhibit Gal-8-induced apoptosis of Th17 cells. *In vitro* differentiated Th17 cells from IL-17A-GFP reporter mice were purified based on IL-17A expression (GFP+) and incubated with Gal-8 (20 μg/ml) in the presence of lactose, sucrose or anti-Gal-8 antibodies affinity purified from pooled serum of MS patients. The extent of apoptosis was quantified as the frequency of Annexin V+ 7AAD+ cells of the sample relative to the frequency of Annexin V+ 7AAD+ cells of the untreated control. Representative contour plots are shown in upper panels. Quantification of a representative experiment is shown in the lower panel. Values represent mean + SEM of triplicates. Data from a representative from four independent experiments is shown. **, p<0.01; ***, p < .001 by one-way ANOVA followed by Tukey’s post-hoc test.

To evaluate whether MS patients generate function-blocking anti-Gal-8 autoantibodies we performed two assays. The established assay of cell adhesion to Gal-8-coated coverslips, which assesses glycan-mediated interaction of Gal-8 with integrins [37, 38], showed that anti-Gal-8(+) serum from RRMS patients decreases the adhesion of peripheral blood mononuclear cells (PBMC) (Fig 8A). In addition, we tested the potential of MS-generated anti-Gal-8 antibodies to counteract the apoptotic effect of Gal-8 on activated Th17 cells showed above in Fig 5B. Affinity purified anti-Gal-8 antibodies from a pooled anti-Gal-8(+) sera effectively decreased the apoptosis rate of activated Th17 cells incubated with Gal-8 (Fig 8B). These results indicate that patients with RRMS generate function-blocking anti-Gal-8 antibodies, which have the potential to neutralize the immunosuppressive role of Gal-8.

### Circulating anti-Gal-8 antibodies are associated with worse prognosis in patients with RRMS

As the presence of anti-Gal-8 neutralizing antibodies might mimic the condition of Gal-8 silencing that exacerbates EAE, we next explored the impact of these antibodies on the clinical course of MS. We studied 58 patients, 36 with recent diagnosis of RRMS and 22 with a progressive disease (8 with SPMS and 14 with PPMS). The results show that 33% (19/58) of these patients have anti-Gal-8 antibodies. Interestingly, 90% of the patients bearing Gal-8(+) sera corresponded to the RRMS phenotype. Within the group of RRMS patients, 17 out of 36 had Gal-8 autoantibodies (47%). In contrast, only 9% of patients with progressive forms (2/22) had anti-Gal-8 autoantibodies (p = 0.006, Yates chi-square corrected p value = 0.006. Odds ratio = 8.947; 95% confidence interval = 1.817–44.054) comparing RRMS versus progressive MS) (Table 3). We previously reported a similar Gal-8 antibody frequency of about 10% in healthy individuals [39]. Therefore, even though anti-Gal-8 autoantibodies are not specific for MS [39], their presence associated with RRMS and not with progressive MS phenotypes.

**Table 3:** Frequency of anti-Gal-8 autoantibodies in 58 patients with multiple sclerosis according to relapsing-remitting (RRMS) or progressive forms.

	Multiple Sclerosis	
Autoantibodies	Relapsing-Remitting	Progressive
	n = 36	n = 22
**anti-Gal-8 (+)**	17 (47%)	2 (10%)
**anti-Gal-8 (-)**	19 (53%)	20 (90%)

Patients with RRMS had higher frequency of anti-Gal-8 antibodies than progressive forms. Yates chi-square corrected p value = 0.006. Odds ratio = 8.947 (95% confidence interval = 1.817–44.054).

**Fig 9:**
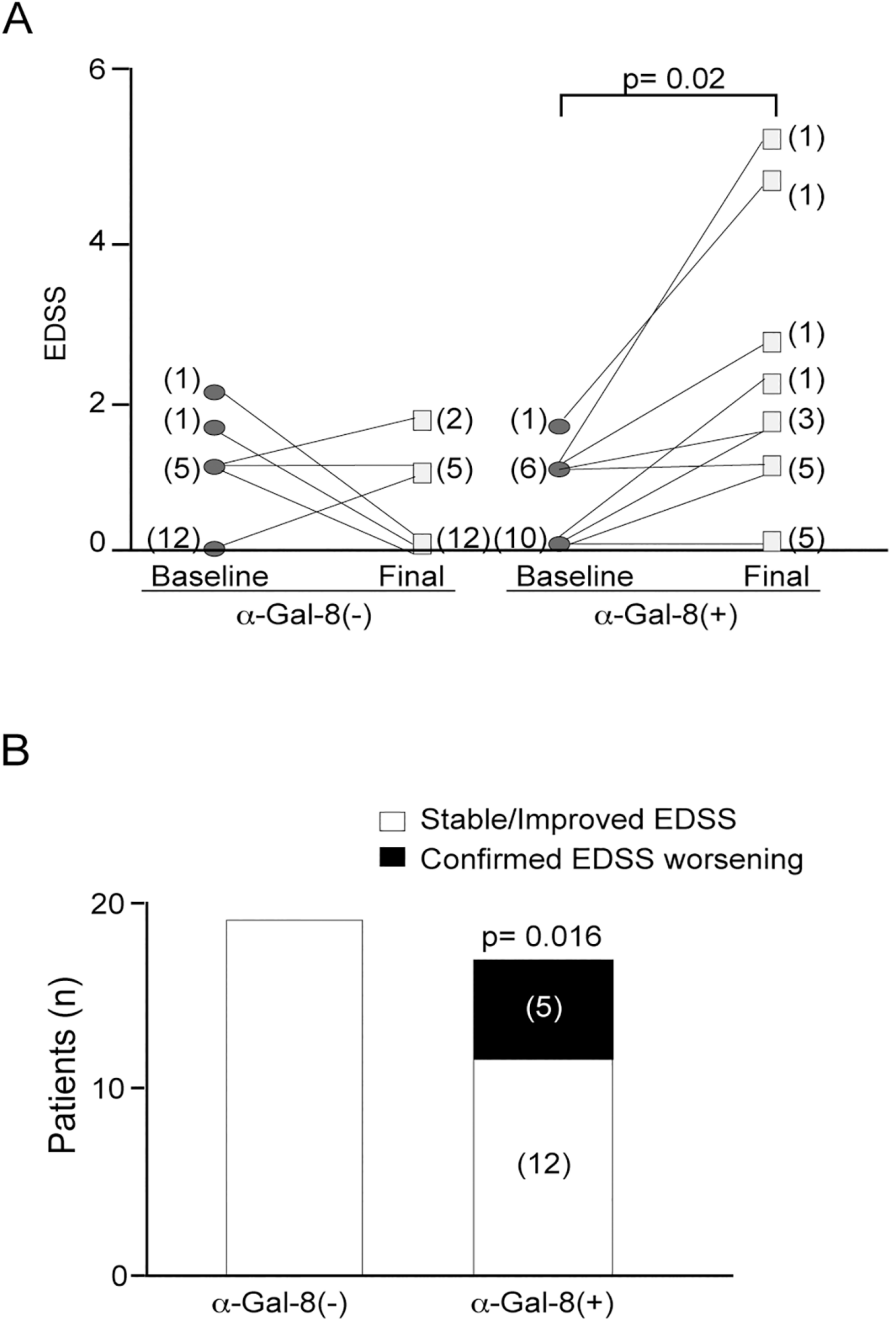
Anti-Gal-8 autoantibodies correlate with worse disability scores in RRMS patients. (A) RRMS patients with and without autoantibodies were followed during an average of 12 months. Patients (n = 17) with anti-Gal-8(+) sera have worse EDSS at the end of follow-up than patients (n = 19) without anti-Gal-8 autoantibodies (mean EDSS 1.5 vs 0, *p = 0.002, nonparametric Mann-Whitney U test), independent of the treatment received or number of relapses during this period. (B) At the end of follow-up, 5/17 patients with anti-Gal-8 autoantibodies developed confirmed EDSS worsening vs 0/19 of patients without anti-Gal-8 autoantibodies (*p = 0.016 by Fisher test).

We next analyzed whether anti-Gal-8 antibodies associate with worse prognosis in RRMS patients. Before starting the DMD treatment, RRMS patients with or without Gal-8 autoantibodies showed no differences in age, gender, age at onset, disease duration, EDSS, and presence of baseline gadolinium-enhanced T1 lesions (S2 Table). However, clear differences appeared during an average of 12 months of follow-up. EDSS worsening occurred with higher frequency in anti-Gal-8(+) patients, independently of treatment and relapse number (Fig 9A; Table 4; mean EDSS 1.5 vs 0, p = 0.02). Five out of 17 patients with anti-Gal-8(+) sera had clinically relevant EDSS worsening, while none of the anti-Gal-8(-) patients worsened during this follow-up period (Fig 9B; p = 0.016).

**Table 4:** Different EDSS outcome in RRMS patients according to the presence of anti-Gal-8 autoantibodies.

Relapsing-Remitting Multiple Sclerosis	Anti-Gal-8 (+) n = 17	Anti-Gal-8 (-) n = 19	Mann-Whitney U test p Value
Disease Duration (years) since first symptoms to baseline; Median (range)	2.0 (1–16)	3.0 (1–9)	0.54
Follow-up (years) since baseline to last assessment Median (range)	1.1 (0.1–1.8)	1.8 (0.1–2.4)	0.62
Annualized relapse rate Median (range)	0.8 (0.1–1.7)	0.6 (0.2–1.9)	0.24
Baseline EDSS (Scale 0–10); Median (range)	0.0 (0.0–1.5)	0.0 (0.0–2.0)	0.93
Final EDSS (Scale 0–10); Median (range)	1.5 (0.0–5.0)	0 (0.0–1.5)	0.02

RRMS: Relapsing-remitting multiple sclerosis; SD: Standard deviation. Mann-Whitney U test. Evolution is independent of relapse number and associates with the presence of anti-Gal-8 autoantibodies. Patients with anti-Gal-8 autoantibodies had worse EDSS.

Considering our previous results in mice, the association of anti-Gal-8 antibodies with severity in RRMS patients might be due to an interference with the immunosuppressive role of Gal-8. Within the RRMS group of patients these antibodies constitute a potential early prognostic marker.

## Discussion

Our results demonstrate a crucial role of Gal-8 and its function-blocking antibodies in the pathogenesis of MS. We first show that Gal-8 exerts an immunosuppressive protective influence against EAE, as revealed by both the enhanced disease developed in Gal-8 KO mice and the ameliorating effect of Gal-8 treatment. The mechanism likely involves a modulating action on the balance of Th1 and Th17 cell polarization and differentiation of their respective CXCR3^+^ and CCR6^+^ Tregs. We then show that patients with MS generate function-neutralizing Gal-8 antibodies that can counteract the immunosuppressive Gal-8 function. Our clinical analysis disclosed an association of circulating anti-Gal-8 antibodies with worse evolution in newly diagnosed and still untreated RRMS patients, showing EDSS worsening within the first year of follow up. Thus, anti-Gal-8 antibodies emerge as a potential early prognostic biomarker.

We show that Gal-8 KO mice at steady state display increased CXCR3^+^ Tregs and decreased CCR6^+^ Tregs frequencies, developing exacerbated EAE accompanied by an increased polarization toward Th17 cells after MOG immunization. *In vitro*, Gal-8 reduces the Th17 cell response to re-stimulation with MOG only in splenocytes from MOG-immunized Gal-8 KO but not from wild-type mice. Th1 cells are not affected. This suggests that Gal-8 eliminates activated Th17 but not Th1 cells during *in vivo* immunization. In congruency, we found that Gal-8 induces apoptosis of anti-CD3/anti-CD28 activated Th17 cells *in vitro*. The relative contribution of Th1 versus Th17 cells in MS pathogenesis remains contentious [3]. The Th17/Th1 ratio likely influences the inflammatory regions in the CNS [48]. Dysregulation of Th17 cells seems to be a main driver of inflammation [49–51], while Th1 lymphocytes producing IFN-γ can play either inflammatory or protective roles [52–54]. Although there is evidence indicating that Th17 cells predominate over Th1 cells in EAE [1, 49, 50, 55], other studies suggest that both Th1 and Th17 cells can drive autoimmune-mediated CNS pathology [48, 56]. Our results of Th17 polarization together with the alterations in CXCR3^+^ and CCR6^+^ Tregs, configuring an immunologic context prone to develop an exacerbated EAE, support the notion that both Th17 and Th1 functions are compromised in the pathogenesis of this disease.

An immunosuppressive role for Gal-8 has been originally suggested by its apoptotic effect on Jurkat T cells and CD3/CD28-activated human peripheral T cells [29]. Such an apoptotic effect involved a little known signaling pathway, in which Gal-8 increases phosphatidic acid leading to ERK activation and phosphodiesterase-4-mediated down regulation of protein kinase A [29]. More recently, Gal-8 was shown to kill *in vitro* differentiated Th17 cells, as we corroborated here in a different setting, and to promote the differentiation of Tregs with ameliorating effects on EAU [30]. In a mouse model of autoimmune uveitis involving exacerbated Th1 and Th17 activities and Treg dysfunction, Sampson et al. [30] showed that Gal-8 treatment increased the differentiation of Tregs at sites of ongoing inflammation, such as draining lymph nodes and retina, but not systemically, as the spleen CD4^+^ T cell subpopulations remained unchanged. These Gal-8-polarized Tregs expressed CTL-4 and IL-10 markers of immune suppression activity and most of them lacked neurophilin expression, suggesting a peripheral rather than thymic origin [30]. Gal-8 also promoted the differentiation of Tregs with high immunosuppressive properties from splenic CD4+ T-cells *in vitro*, modulating the known roles of IL-2 and TGF-β receptors in this process [31]. These acute effects most likely contribute to ameliorate the severity of autoimmune uveitis in mice treated with Gal-8 [30], as well as EAE in our present experiments.

However, our results reveal a more complex role of Gal-8 on Treg homeostasis than previously appreciated from studies showing Gal-8-promoted Treg differentiation [30, 31]. At steady state, Gal-8 KO mice have lower frequency of CCR6+ Tregs, which are suppressors of Th17-mediated inflammation [8]. This condition, together with increased Th17 cells, probably provides an important background for the exacerbated EAE developed by these animals. Endogenous Gal-8 could be required for appropriate differentiation of these particular Tregs. However, the chronic deficit of Gal-8 function in KO mice clearly configures a more complex condition. Instead of resulting in a lower Treg polarization, Gal-8 KO mice have increased total Tregs, mostly composed of CXCR3^+^Tregs, which should compromise Th1-mediated functions [8]. Such Treg imbalance might originate at the thymus where Gal-8 has been shown to be expressed and can induce thymocyte apoptosis [57]. Indeed, a deficit in the reported Gal-8-modulation of IL-2 and TGF-β receptors [31], crucial for Treg differentiation and many other immune processes [6, 58], might underlie this alteration. The exacerbated EAE of Gal-8 KO mice and the expression of CXCR3 in the FoxP3^+^ T cell population suggest a Treg polarization distinct from the immunosuppressive-competent Tregs differentiated under a Gal-8 environment [30, 31]. In the absence of Gal-8, Treg polarization might mimic the immunosuppressive-defective and proinflammatory CXCR3^+^ Tregs found enriched in RRMS patients [59]. Interestingly, Th1-suppressive CXCR3^+^ Tregs play protective roles under transient inflammatory stimuli [9, 10, 60], but are reprogrammed to effector Th1 cells producing IFN-γ with inflammatory activity during sustained or chronic inflammation [8, 60]. IFN-γ, the hallmark Th1 cytokine, can promote both protective and pathogenic roles in EAE and MS [52–54]. RRMS patients display a higher frequency of Tregs with an IFN-γ-expressing Th1-like phenotype that has reduced suppressive activity [59]. Furthermore, IFN-β, a first-line DMD therapy for RRMS returns the frequency of Th1-like CXCR3^+^ Tregs to that of healthy controls and increases the frequency of Th17 suppressive Tregs [59]. Therefore, the exacerbated EAE developed by Gal-8 KO mice, similarly to RRMS, likely involves dysregulation of Th1-and Th17-suppressive Tregs. Gal-8 might be a master regulator of Treg subpopulations that suppress Th1- and Th17-mediated functions and consequently might modulate the pathogenic effector functions of Th1 and Th17 cells in MS.

The high expression levels of Gal-8 found in the choroid plexus of mouse brain and the presence of Gal-8 in human CSF suggest a direct role of Gal-8 in the CNS immune-surveillance circuit. The immune-surveillance circuit includes subarachnoid regions bathed in CSF and the deep cervical lymph nodes where dura lymphatic vessels drain CSF from the brain [3, 61, 62]. Therefore, Gal-8 present in the CSF might modulate autoimmune inflammatory events at all these locations. In addition, the choroid plexus generates and regulates CSF and constitutes the main gate for T cells to cross the blood-CSF barrier during immune surveillance and the initial stages of EAE [3]. Th17 cells express the receptor CCR6 for the chemokine CCL20, which is constitutively expressed in the choroid plexus and is fundamental for EAE development [63]. Whether Gal-8 interacts with CCR6 remains unknown. However, CNS homing of MS- and EAE-pathogenic Th1 and Th17 cells also depends on α4β1 integrin and LFA-1 integrins, respectively [48, 64–66]. Gal-8 binds poorly to α4β1 integrin [37], but effectively binds to LFA-1 and inhibits its interaction with ICAM [38]. It is thus possible that Gal-8 produced in the choroid plexus restricts CNS homing mainly of Th17 cells, the first T cell subset that migrates into the CNS during EAE [3].

In addition, our results suggest that an impaired Gal-8 immunosuppressive role in MS, mimicking the autoimmune CNS enhanced condition of Gal-8 KO mice, might occur within an autoimmune context that generates anti-Gal-8 neutralizing antibodies similar to those described in SLE [29, 37–39]. Our early studies showed that Gal-8-induced apoptosis of Jurkat cells can be counteracted by anti-Gal-8 antibodies from lupus patients, thus suggesting for the first time a role of these antibodies in autoimmunity [29]. Here we detected anti-Gal-8 antibodies in both the serum and CSF of MS patients. The circulating anti-Gal-8 antibodies obtained from MS patients blocked cell adhesion to Gal-8 and also the apoptotic effect of Gal-8 upon activated Th17 cells. Our clinical analysis suggests a pathogenic role of these antibodies. Anti-Gal-8 antibodies detected in serum associated with RRMS and not with the progressive forms of the disease. This might reflect differences in the autoimmune status between the different stages and phenotypes of the disease [1, 3] and could help to precisely diagnose uncertain cases. More importantly, these antibodies predict RRMS prognosis. The clinical course of RRMS is heterogeneous, varying from few relapses and low physical burden to rapid accumulation of disability [2]. As shown by clinically relevant EDSS worsening, RRMS patients bearing circulating Gal-8 antibodies at the moment of diagnosis have higher probability of disability progression within an evolution period of 12 months. Despite the small sample size and low EDSS scores, our data reached statistical significance associating clinical disability progression with anti-Gal-8 antibodies. The experimental evidence of an immunesuppressive role of Gal-8 in EAE provides robust mechanistic rational to these clinical findings. Therefore, MS patients can generate function-blocking anti-Gal-8 antibodies with pathogenic potential, as they can neutralize the immunosuppressive role of endogenous Gal-8 that would normally ameliorate CNS inflammation. Biomarkers that predict higher probability of developing disability at the moment of MS diagnosis, thus helping to optimize a personalized therapy, have long been pursued and are still lacking [67, 68]. Our results open a new possibility pointing to Gal-8 antibodies as suitable serum biomarkers for worse prognosis in recently diagnosed RRMS patients. Indeed, such an important possibility would require further validation in a larger cohort of patients.

A pathogenic role of antibodies in MS pathogenesis has long been debated [14]. Against such a role are the rapid responses elicited by B-cell depleting therapy almost without affecting antibody levels [3, 14]. B-cell functions such as antigen presentation and immune regulation independent of antibody production have been involved in MS evolution [69]. On the other hand, intrathecal production of oligoclonal bands, clonal expansion of B cells coinciding in CSF and lesions, detection of antibodies and complement deposition in CNS lesions, the presence of follicle-like aggregates in the meninges and the beneficial effects of plasmapheresis in some patients are all circumstantial evidence supporting a pathogenic role of antibodies [3, 14]. The identification of clinically relevant antigens is still a major problem [14]. MS patients generate autoantibodies against neurons, oligodendrocytes, astrocytes and immune cell-specific antigens, encompassing a wide range of cell surface and intracellular molecules, including proteins, lipids, glycans, gangliosides and DNA [70]. Studies on relevant targets have been focused on myelin and axonal elements that might mediate demyelination and neurodegeneration [14, 71]. The most studied antibodies against MOG [72, 73], two components of the node of Ranvier, neurofascin [74] and contactin [75] and the potassium channel KIR4.1, expressed in glial cells [76, 77], have experimentally demonstrated pathogenic potential but so far do not correlate with clinical evolution [14]. Oligoclonal IgM bands against myelin lipids present in CSF predict aggressive evolution of RRMS [78–81], but their extensive use as biomarkers seems limited by methodological difficulties [71, 82]. Our results point instead to a circulating antibody compromising the function of an immune suppressor factor, such as Gal-8, involved in the Th17, Th1 and Treg cell homeostasis. Neutralizing Gal-8 antibodies might underlie risk conditions for worse RRMS evolution counteracting Gal-8-mediated immune-modulating functions.

Several endogenous galectins probably contribute to the attenuation of autoimmune-inflammation in the CNS through both redundant and complementary pathways. Gal-1- and Gal-9-deficient mice develop more severe EAE due to selective expansion of antigen-specific Th1 and Th17 cells and to increased immunogenicity of dendritic cells [20, 83]. In contrast, Gal-3 deficiency reduces the severity of EAE [26]. The fact that mice lacking Gal-1, Gal-9 or Gal-8 all develop more severe EAE suggests that the remaining galectins cannot compensate for the absence of any of the others. Fine variations in their CRD preference for galactose-containing oligosaccharides can underlie complementary roles of these lectins [19–22]. The N- terminal CRD of Gal-8 has a unique high affinity for α2,3-sialylated glycans [27, 28]. Therefore, Gal-1, Gal-9 and Gal-8 can sustain immune-suppressive conditions sensitive to disruption by the absence or decreased function of any of them.

Galectins also offer new therapeutic opportunities. Gal-1 and Gal-9 enhance the frequency and immunosuppressive capacity of Treg cells [84, 85] and reduce EAE [20, 86]. Gal-1 also attenuates EAE severity decreasing the activation of microglia [25]. As we show here, Gal-8 treatment ameliorates EAE and might be especially indicated to counteract the severity in RRMS patients bearing Gal-8 function-blocking antibodies. It would be interesting to assess whether RRMS patients also generate neutralizing antibodies against Gal-1 and Gal-9 associated with worse prognosis.

In conclusion, our results point to Gal-8 as an endogenous immunosuppressor that limits the autoimmune attack on the CNS, which can be blocked by antibodies leading to worse prognosis. Our clinical results together with a plausible pathogenic mechanism suggest that circulating anti-Gal-8 antibodies might be an early prognostic maker in RRMS and prompt to consider a therapeutic use of this lectin in patients bearing anti-Gal-8 antibodies.

## Supporting information

S1 Table Gal-8 expression revealed by LacZ histochemistry in the mouse brain. Brain sections of the heterocygous knock-in *Lgals8*^+/-^ mice with positive reaction for β-gal histochemistry are listed as regions considered to express Gal-8.
(PDF)

S2 Table Baseline characteristics of RRMS patients with and without anti-Gal-8 autoantibodies. RRMS patients before starting the DMD treatment, with or without Gal-8 autoantibodies in sera, share similar characteristics of age, gender, age at onset, disease duration, EDSS, and presence of baseline gadolinium-enhanced T1 lesions.
(PDF)

S1 File Raw data of Fig 1 and Table 1. Daily clinical score of EAE Lgals8^+/+^ versus Lgals8^-/-^ mice, induced by immunization of an emulsion containing 150 μg of MOG35-55 peptide (MOGp) in 8-12-week-old Lgals8^-/-^ and Lgals8^+/+^ mice. Daily monitored clinical scale of EAE symptoms: 0, no clinical signs; 1, loss of tail tone; 2, flaccid tail; 3, incomplete paralysis of one or two hind legs; 4, complete hind limb paralysis; 5, moribund 6, death. Data was used to establish daily progression of EAE (Fig 1) and clinical parameters in Table 1.
(PDF)

S2 File Raw data of Fig 2. Fig 2A: FACS-analyzed frequencies of immune cell subpopulations in splenocytes from 8-12-week-old female Lgals8^+/+^ (WT) and Lgals8^-/-^ (KO) mice. Table 2A: Frequencies of T cells (CD4^+^), B cells (CD19^+^), CD8^+^ T cells and dendritic cells (CD11c^+^). Table 2B: CD4^+^ T cell subpopulations Th1, Th2 and Th17. For polyclonal T cell activation, splenocytes were grown in the presence of 1 μg/ml of αCD3 /μCD28 antibodies for 72 h. For the last 4 h of culture cell were stimulated incubating with 50 ng/ml PMA, 500 ng/ml ionomycin, and 10 μg/ml brefeldin A. Tables show frequencies for Th1, Th2 and Th17 in Lgals8^+/+^ (WT) and Lgals8^-/-^ (KO) mice in untreated condition (UN) or under polyclonal activation (aCD3/28). Fig 2B: Splenocytes isolated from *Lgals8*+/+ (WT) and *Lgals8*^-/-^ (KO) mice analyzed by FACS: (A) Dendritic cells (CD11c^+^), B cells (CD19^+^), CD8^+^ T cells and different CD4^+^ T cells subsets, naïve (CD44^-^CD62L^+^), effector (CD44^+^CD62L^+^), memory (CD44+CD62L^-^) and total cells analyzed in the subset of viable CD4^+^ CD25^-^ T cells. Results from 4–8 independent experiments show that Gal-8 deficit favors selective Th17 cell differentiation upon polyclonal activation.
(PDF)

S3 File Raw data of Fig 3: Frequencies of Th1 and Th17 subpopulations in splenocytes from 10 day-induced EAE Lgals8^-/-^ (KO) and Lgals8^+/+^ (WT) mice re-stimulated for 72 h with MOGp in the absence (UN) or presence of Gal-8. (PDF)

S4 File Raw data of Fig 4. Splenocytes isolated from *Lgals8*^+/+^ (WT) and *Lgals8*^-/-^ (KO) mice analyzed at steady state for total Tregs (Foxp3^+^), CXCR3^+^ and CCR6^+^ frequency in the Treg (Foxp3^+^ CD4^+^) population. Upper Table: Total Tregs obtained from five WT and five Gal8-KO mice (upper table). Middle Table: CXCR3^+^CCR6^-^. Bottom Table: CXCR3^-^CCR6^+^. Analysis from three WT and three Gal8-KO mice. Data show that galectin-8 deficit increases the frequency of total Tregs and CXCR3^+^ Tregs.
(PDF)

S5 File Raw data of Fig 5 and Table 2: Daily clinical score of EAE Gal-8-treated versus vehicle–treated WT mice. EAE was induced in wild-type C57BL/6J mice and simultaneously treated (i.p.) with either 100 μg recombinant Gal-8 or PBS (control group) during 20 consecutive days. EAE symptoms monitored daily using the following scale: 0, no clinical signs; 1, loss of tail tone; 2, flaccid tail; 3, incomplete paralysis of one or two hind legs; 4, complete hind limb paralysis; 5, moribund 6, death.
(PDF)

S6 File Raw data of Fig 8A. Number of adherent cells counted in six randomly selected fields after incubation with anti-Gal-8 autoantibodies from three positive (MS10, MS14 and MS20) and three negative patients (MS 21, MS 18 and MS27). Anti-Gal-8 autoantibodies inhibit cell adhesion.
(PDF)

S7 File Raw data of Fig 8B. *In vitro* differentiated Th17 cells from IL-17A-GFP reporter mice were purified based on IL-17A expression (GFP+) and incubated with Gal-8 (20 μg/ml) in the presence of lactose, sucrose or anti-Gal-8 antibodies affinity purified from pooled serum of MS patients. Numbers are the frequency of Annexin V^+^ 7AAD^+^ cells of the treated sample relative to the frequency of Annexin V^+^ 7AAD^+^ cells of untreated control, representing apoptosis, from three independent experiments. Anti-Gal-8 autoantibodies inhibit Gal-8-induced apoptosis of Th17 cells.
(PDF)

S8 File Raw data of Patients. Clinical characteristic of 17 RRMS patients anti-Gal-8(+) and 19 RRMS patients anti-Gal-8(-): N (number of patient); Gender (1 is man and 0 is woman); Age (is age at Anti-Gal-8 assay in years); Age at onset MS (age at first symptoms in years); Diagnostic delay (time between age of onset and age of MS diagnosis); Disease duration (years between onset of MS and age at sample assay); Basal EDSS (EDSS at moment of Anti-Gal assay); Brain MRI Gd^+^ (number of Gadolinium enhancement T1 lesions at brain at moment of Anti-Gal assay); Spinal MRI Gd+ (number of Gadolinium enhancement T1 lesions at spinal cord at moment of Anti-Gal assay); DMD treatment (DMD treatment after Anti-Gal assay); Final EDSS (EDSS at follow-up); ARR (annual relapses rate in follow-up); Follow-up (years of follow-up after anti-Gal-8 assay).
(PDF)

## References

[1] Compston A, Coles A. Multiple sclerosis. Lancet. 2008;372(9648):1502–17. doi: 10.1016/S0140-6736(08)61620-7 . 18970977 10.1016/S0140-6736(08)61620-7

[2] Harrison DM. Multiple sclerosis. Annals of internal medicine. 2014;160(7):ITC4-2-ITC4-18; quiz ITC4-6. doi: 10.7326/0003-4819-160-7-201404010-01004 . 24763702 10.7326/0003-4819-160-7-201404010-01004

[3] Dendrou CA, Fugger L, Friese MA. Immunopathology of multiple sclerosis. Nature reviews Immunology. 2015;15(9):545–58. doi: 10.1038/nri3871 . 26250739 10.1038/nri3871

[4] Rieckmann P, Smith KJ. Multiple sclerosis: more than inflammation and demyelination. Trends in neurosciences. 2001;24(8):435–7. . 11488295 10.1016/s0166-2236(00)01860-9

[5] Fletcher JM, Lalor SJ, Sweeney CM, Tubridy N, Mills KH. T cells in multiple sclerosis and experimental autoimmune encephalomyelitis. Clinical and experimental immunology. 2010;162(1):1–11. doi: 10.1111/j.1365-2249.2010.04143.x ; PubMed Central PMCID: PMCPMC2990924. 20682002 10.1111/j.1365-2249.2010.04143.x PMC2990924

[6] Kleinewietfeld M, Hafler DA. Regulatory T cells in autoimmune neuroinflammation. Immunol Rev. 2014;259(1):231–44. doi: 10.1111/imr.12169 ; PubMed Central PMCID: PMCPMC3990868. 24712469 10.1111/imr.12169 PMC3990868

[7] Dhaeze T, Stinissen P, Liston A, Hellings N. Humoral autoimmunity: a failure of regulatory T cells? Autoimmun Rev. 2015;14(8):735–41. doi: 10.1016/j.autrev.2015.04.006 . 25913138 10.1016/j.autrev.2015.04.006

[8] Campbell DJ, Koch MA. Phenotypical and functional specialization of FOXP3+ regulatory T cells. Nature reviews Immunology. 2011;11(2):119–30. doi: 10.1038/nri2916 ; PubMed Central PMCID: PMCPMC3289970. 21267013 10.1038/nri2916 PMC3289970

[9] Koch MA, Tucker-Heard G, Perdue NR, Killebrew JR, Urdahl KB, Campbell DJ. The transcription factor T-bet controls regulatory T cell homeostasis and function during type 1 inflammation. Nature immunology. 2009;10(6):595–602. doi: 10.1038/ni.1731 ; PubMed Central PMCID: PMCPMC2712126. 19412181 10.1038/ni.1731 PMC2712126

[10] Hall AO, Beiting DP, Tato C, John B, Oldenhove G, Lombana CG, et al The cytokines interleukin 27 and interferon-γ promote distinct Treg cell populations required to limit infection-induced pathology. Immunity. 2012;37(3):511–23. doi: 10.1016/j.immuni.2012.06.014 ; PubMed Central PMCID: PMCPMC3477519. 22981537 10.1016/j.immuni.2012.06.014 PMC3477519

[11] Chaudhry A, Rudra D, Treuting P, Samstein RM, Liang Y, Kas A, et al CD4+ regulatory T cells control TH17 responses in a Stat3-dependent manner. Science. 2009;326(5955):986–91. doi: 10.1126/science.1172702 ; PubMed Central PMCID: PMCPMC4408196. 19797626 10.1126/science.1172702 PMC4408196

[12] Muller M, Carter SL, Hofer MJ, Manders P, Getts DR, Getts MT, et al CXCR3 signaling reduces the severity of experimental autoimmune encephalomyelitis by controlling the parenchymal distribution of effector and regulatory T cells in the central nervous system. Journal of immunology. 2007;179(5):2774–86. . 17709491 10.4049/jimmunol.179.5.2774

[13] Yamazaki T, Yang XO, Chung Y, Fukunaga A, Nurieva R, Pappu B, et al CCR6 regulates the migration of inflammatory and regulatory T cells. Journal of immunology. 2008;181(12):8391–401. ; PubMed Central PMCID: PMCPMC2752441. 19050256 10.4049/jimmunol.181.12.8391 PMC2752441

[14] Probstel AK, Sanderson NS, Derfuss T. B Cells and Autoantibodies in Multiple Sclerosis. International journal of molecular sciences. 2015;16(7):16576–92. doi: 10.3390/ijms160716576 ; PubMed Central PMCID: PMCPMC4519967. 26197319 10.3390/ijms160716576 PMC4519967

[15] Rabinovich GA, Croci DO. Regulatory circuits mediated by lectin-glycan interactions in autoimmunity and cancer. Immunity. 2012;36(3):322–35. Epub 2012/03/27. doi: 10.1016/j.immuni.2012.03.004 . 22444630 10.1016/j.immuni.2012.03.004

[16] Elola MT, Blidner AG, Ferragut F, Bracalente C, Rabinovich GA. Assembly, organization and regulation of cell-surface receptors by lectin-glycan complexes. The Biochemical journal. 2015;469(1):1–16. doi: 10.1042/BJ20150461 . 26173257 10.1042/BJ20150461

[17] Kaltner H, Gabius HJ. A toolbox of lectins for translating the sugar code: the galectin network in phylogenesis and tumors. Histol Histopathol. 2012;27(4):397–416. Epub 2012/03/01. doi: 10.14670/HH-27.397 22374719 10.14670/HH-27.397

[18] Nabi IR, Shankar J, Dennis JW. The galectin lattice at a glance. J Cell Sci. 2015;128(13):2213–9. doi: 10.1242/jcs.151159 . 26092931 10.1242/jcs.151159

[19] Hirabayashi J, Hashidate T, Arata Y, Nishi N, Nakamura T, Hirashima M, et al Oligosaccharide specificity of galectins: a search by frontal affinity chromatography. Biochim Biophys Acta. 2002;1572(2–3):232–54. Epub 2002/09/12. . 12223272 10.1016/s0304-4165(02)00311-2

[20] Toscano MA, Bianco GA, Ilarregui JM, Croci DO, Correale J, Hernandez JD, et al Differential glycosylation of TH1, TH2 and TH-17 effector cells selectively regulates susceptibility to cell death. Nat Immunol. 2007;8(8):825–34. Epub 2007/06/26. doi: 10.1038/ni1482 . 17589510 10.1038/ni1482

[21] Stowell SR, Arthur CM, Mehta P, Slanina KA, Blixt O, Leffler H, et al Galectin-1, -2, and -3 exhibit differential recognition of sialylated glycans and blood group antigens. J Biol Chem. 2008;283(15):10109–23. Epub 2008/01/25. doi: 10.1074/jbc.M709545200 . 18216021 10.1074/jbc.M709545200 PMC2442294

[22] Zhuo Y, Chammas R, Bellis SL. Sialylation of beta1 integrins blocks cell adhesion to galectin-3 and protects cells against galectin-3-induced apoptosis. J Biol Chem. 2008 Epub 2008/06/14. doi: 10.1074/jbc.M800015200 . 10.1074/jbc.M8000015200 PMC2494929 18676377

[23] Steelman AJ, Smith R, 3rd, Welsh CJ, Li J. Galectin-9 protein is up-regulated in astrocytes by tumor necrosis factor and promotes encephalitogenic T-cell apoptosis. J Biol Chem. 2013;288(33):23776–87. doi: 10.1074/jbc.M113.451658 ; PubMed Central PMCID: PMC3745324. 23836896 10.1074/jbc.M113.451658 PMC3745324

[24] Zhu C, Anderson AC, Schubart A, Xiong H, Imitola J, Khoury SJ, et al The Tim-3 ligand galectin-9 negatively regulates T helper type 1 immunity. Nat Immunol. 2005;6(12):1245–52. Epub 2005/11/16. doi: 10.1038/ni1271 . 16286920 10.1038/ni1271

[25] Starossom SC, Mascanfroni ID, Imitola J, Cao L, Raddassi K, Hernandez SF, et al Galectin-1 deactivates classically activated microglia and protects from inflammation-induced neurodegeneration. Immunity. 2012;37(2):249–63. Epub 2012/08/14. doi: 10.1016/j.immuni.2012.05.023 ; PubMed Central PMCID: PMC3428471. 22884314 10.1016/j.immuni.2012.05.023 PMC3428471

[26] Jiang HR, Al Rasebi Z, Mensah-Brown E, Shahin A, Xu D, Goodyear CS, et al Galectin-3 deficiency reduces the severity of experimental autoimmune encephalomyelitis. J Immunol. 2009;182(2):1167–73. Epub 2009/01/07. . 19124760 10.4049/jimmunol.182.2.1167

[27] Ideo H, Matsuzaka T, Nonaka T, Seko A, Yamashita K. Galectin-8-N-domain recognition mechanism for sialylated and sulfated glycans. J Biol Chem. 2011;286(13):11346–55. doi: 10.1074/jbc.M110.195925 ; PubMed Central PMCID: PMCPMC3064191. 21288902 10.1074/jbc.M110.195925 PMC3064191

[28] Ideo H, Seko A, Ishizuka I, Yamashita K. The N-terminal carbohydrate recognition domain of galectin-8 recognizes specific glycosphingolipids with high affinity. Glycobiology. 2003;13(10):713–23. Epub 2003/07/10. doi: 10.1093/glycob/cwg094 . 12851289 10.1093/glycob/cwg094

[29] Norambuena A, Metz C, Vicuna L, Silva A, Pardo E, Oyanadel C, et al Galectin-8 induces apoptosis in Jurkat T cells by phosphatidic acid-mediated ERK1/2 activation supported by protein kinase A down-regulation. J Biol Chem. 2009;284(19):12670–9. Epub 2009/03/12. doi: 10.1074/jbc.M808949200 . 19276072 10.1074/jbc.M808949200 PMC2675996

[30] Sampson JF, Hasegawa E, Mulki L, Suryawanshi A, Jiang S, Chen WS, et al Galectin-8 Ameliorates Murine Autoimmune Ocular Pathology and Promotes a Regulatory T Cell Response. PLoS One. 2015;10(6):e0130772 Epub 2015/07/01. doi: 10.1371/journal.pone.0130772 ; PubMed Central PMCID: PMC4488339. 26126176 10.1371/journal.pone.0130772 PMC4488339

[31] Sampson JF, Suryawanshi A, Chen WS, Rabinovich GA, Panjwani N. Galectin-8 promotes regulatory T-cell differentiation by modulating IL-2 and TGFbeta signaling. Immunol Cell Biol. 2016;94(2):213–9. doi: 10.1038/icb.2015.72 ; PubMed Central PMCID: PMCPMC4747822. 26282995 10.1038/icb.2015.72 PMC4747822

[32] Nishi N, Shoji H, Seki M, Itoh A, Miyanaka H, Yuube K, et al Galectin-8 modulates neutrophil function via interaction with integrin αM. Glycobiology. 2003;13(11):755–63. doi: 10.1093/glycob/cwg102 12881409 10.1093/glycob/cwg102

[33] Eshkar Sebban L, Ronen D, Levartovsky D, Elkayam O, Caspi D, Aamar S, et al The involvement of CD44 and its novel ligand galectin-8 in apoptotic regulation of autoimmune inflammation. Journal of immunology. 2007;179(2):1225–35. . 17617615 10.4049/jimmunol.179.2.1225

[34] Ruiz FM, Scholz BA, Buzamet E, Kopitz J, Andre S, Menendez M, et al Natural single amino acid polymorphism (F19Y) in human galectin-8: detection of structural alterations and increased growth-regulatory activity on tumor cells. FEBS J. 2014;281(5):1446–64. Epub 2014/01/15. doi: 10.1111/febs.12716 . 24418318 10.1111/febs.12716

[35] Zhang S, Moussodia RO, Vertesy S, Andre S, Klein ML, Gabius HJ, et al Unraveling functional significance of natural variations of a human galectin by glycodendrimersomes with programmable glycan surface. Proc Natl Acad Sci U S A. 2015;112(18):5585–90. Epub 2015/04/23. doi: 10.1073/pnas.1506220112 ; PubMed Central PMCID: PMC4426414. 25902539 10.1073/pnas.1506220112 PMC4426414

[36] Pal Z, Antal P, Srivastava SK, Hullam G, Semsei AF, Gal J, et al Non-synonymous single nucleotide polymorphisms in genes for immunoregulatory galectins: association of galectin-8 (F19Y) occurrence with autoimmune diseases in a Caucasian population. Biochim Biophys Acta. 2012;1820(10):1512–8. doi: 10.1016/j.bbagen.2012.05.015 . 22683700 10.1016/j.bbagen.2012.05.015

[37] Carcamo C, Pardo E, Oyanadel C, Bravo-Zehnder M, Bull P, Caceres M, et al Galectin-8 binds specific beta1 integrins and induces polarized spreading highlighted by asymmetric lamellipodia in Jurkat T cells. Exp Cell Res. 2006;312(4):374–86. doi: 10.1016/j.yexcr.2005.10.025 16368432 10.1016/j.yexcr.2005.10.025

[38] Vicuna L, Pardo E, Curkovic C, Doger R, Oyanadel C, Metz C, et al Galectin-8 binds to LFA-1, blocks its interaction with ICAM-1 and is counteracted by anti-Gal-8 autoantibodies isolated from lupus patients. Biological research. 2013;46(3):275–80. doi: 10.4067/S0716-97602013000300008 . 24346075 10.4067/S0716-97602013000300008

[39] Massardo L, Metz C, Pardo E, Mezzano V, Babul M, Jarpa E, et al Autoantibodies against galectin-8: their specificity, association with lymphopenia in systemic lupus erythematosus and detection in rheumatoid arthritis and acute inflammation. Lupus. 2009;18(6):539–46. Epub 2009/04/28. doi: 10.1177/0961203308099973 . 19395456 10.1177/0961203308099973

[40] Valenzuela DM, Murphy AJ, Frendewey D, Gale NW, Economides AN, Auerbach W, et al High-throughput engineering of the mouse genome coupled with high-resolution expression analysis. Nat Biotechnol. 2003;21(6):652–9. Epub 2003/05/06. doi: 10.1038/nbt822 . 12730667 10.1038/nbt822

[41] Poueymirou WT, Auerbach W, Frendewey D, Hickey JF, Escaravage JM, Esau L, et al F0 generation mice fully derived from gene-targeted embryonic stem cells allowing immediate phenotypic analyses. Nat Biotechnol. 2007;25(1):91–9. Epub 2006/12/26. doi: 10.1038/nbt1263 . 17187059 10.1038/nbt1263

[42] Segovia-Miranda F, Serrano F, Dyrda A, Ampuero E, Retamal C, Bravo-Zehnder M, et al Pathogenicity of lupus anti-ribosomal p antibodies: Role of cross-reacting neuronal surface p-antigen in glutamatergic transmission and plasticity. Arthritis & rheumatology. 2015 doi: 10.1002/art.39081 . 25709106 10.1002/art.39081

[43] Prado C, Contreras F, Gonzalez H, Diaz P, Elgueta D, Barrientos M, et al Stimulation of dopamine receptor D5 expressed on dendritic cells potentiates Th17-mediated immunity. Journal of immunology. 2012;188(7):3062–70. doi: 10.4049/jimmunol.1103096 . 22379034 10.4049/jimmunol.1103096

[44] Rowse AL, Naves R, Cashman KS, McGuire DJ, Mbana T, Raman C, et al Lithium controls central nervous system autoimmunity through modulation of IFN-γ signaling. PLoS One. 2012;7(12):e52658 doi: 10.1371/journal.pone.0052658 ; PubMed Central PMCID: PMCPMC3532311. 23285134 10.1371/journal.pone.0052658 PMC3532311

[45] Metz C, Doger R, Riquelme E, Cortes P, Holmes C, Shaughnessy R, et al Galectin-8 promotes migration and proliferation and prevents apoptosis in U87 glioblastoma cells. Biological research. 2016;49(1):33 doi: 10.1186/s40659-016-0091-6 ; PubMed Central PMCID: PMCPMC4962418. 27459991 10.1186/s40659-016-0091-6 PMC4962418

[46] Pardo E, Carcamo C, Massardo L, Mezzano V, Jacobelli S, Gonzalez A, et al [Antibodies against galectin-8 in patients with systemic lupus erythematosus]. Rev Med Chil. 2006;134(2):159–66. Epub 2006/03/24. . 16554922 10.4067/s0034-98872006000200004

[47] Benarroch EE. Choroid plexus—CSF system: Recent developments and clinical correlations. Neurology. 2016;86(3):286–96. doi: 10.1212/WNL.0000000000002298 . 26683646 10.1212/WNL.0000000000002298

[48] Stromnes IM, Cerretti LM, Liggitt D, Harris RA, Goverman JM. Differential regulation of central nervous system autoimmunity by T(H)1 and T(H)17 cells. Nature medicine. 2008;14(3):337–42. doi: 10.1038/nm1715 ; PubMed Central PMCID: PMC2813727. 18278054 10.1038/nm1715 PMC2813727

[49] Langrish CL, Chen Y, Blumenschein WM, Mattson J, Basham B, Sedgwick JD, et al IL-23 drives a pathogenic T cell population that induces autoimmune inflammation. The Journal of experimental medicine. 2005;201(2):233–40. doi: 10.1084/jem.20041257 ; PubMed Central PMCID: PMCPMC2212798. 15657292 10.1084/jem.20041257 PMC2212798

[50] Chen Y, Langrish CL, McKenzie B, Joyce-Shaikh B, Stumhofer JS, McClanahan T, et al Anti-IL-23 therapy inhibits multiple inflammatory pathways and ameliorates autoimmune encephalomyelitis. The Journal of clinical investigation. 2006;116(5):1317–26. doi: 10.1172/JCI25308 ; PubMed Central PMCID: PMCPMC1450386. 16670771 10.1172/JCI25308 PMC1450386

[51] Huber AK, Wang L, Han P, Zhang X, Ekholm S, Srinivasan A, et al Dysregulation of the IL-23/IL-17 axis and myeloid factors in secondary progressive MS. Neurology. 2014;83(17):1500–7. doi: 10.1212/WNL.0000000000000908 ; PubMed Central PMCID: PMC4222856. 25253754 10.1212/WNL.0000000000000908 PMC4222856

[52] Arellano G, Ottum PA, Reyes LI, Burgos PI, Naves R. Stage-Specific Role of Interferon-γ in Experimental Autoimmune Encephalomyelitis and Multiple Sclerosis. Frontiers in immunology. 2015;6:492 doi: 10.3389/fimmu.2015.00492 ; PubMed Central PMCID: PMCPMC4586507. 26483787 10.3389/fimmu.2015.00492 PMC4586507

[53] Naves R, Singh SP, Cashman KS, Rowse AL, Axtell RC, Steinman L, et al The interdependent, overlapping, and differential roles of type I and II IFNs in the pathogenesis of experimental autoimmune encephalomyelitis. Journal of immunology. 2013;191(6):2967–77. doi: 10.4049/jimmunol.1300419 ; PubMed Central PMCID: PMCPMC3779698. 23960239 10.4049/jimmunol.1300419 PMC3779698

[54] Ottum PA, Arellano G, Reyes LI, Iruretagoyena M, Naves R. Opposing Roles of Interferon-Gamma on Cells of the Central Nervous System in Autoimmune Neuroinflammation. Frontiers in immunology. 2015;6:539 doi: 10.3389/fimmu.2015.00539 ; PubMed Central PMCID: PMCPMC4626643. 26579119 10.3389/fimmu.2015.00539 PMC4626643

[55] Kebir H, Ifergan I, Alvarez JI, Bernard M, Poirier J, Arbour N, et al Preferential recruitment of interferon-gamma-expressing TH17 cells in multiple sclerosis. Annals of neurology. 2009;66(3):390–402. doi: 10.1002/ana.21748 . 19810097 10.1002/ana.21748

[56] Kroenke MA, Carlson TJ, Andjelkovic AV, Segal BM. IL-12- and IL-23-modulated T cells induce distinct types of EAE based on histology, CNS chemokine profile, and response to cytokine inhibition. The Journal of experimental medicine. 2008;205(7):1535–41. doi: 10.1084/jem.20080159 ; PubMed Central PMCID: PMCPMC2442630. 18573909 10.1084/jem.20080159 PMC2442630

[57] Tribulatti MV, Mucci J, Cattaneo V, Aguero F, Gilmartin T, Head SR, et al Galectin-8 induces apoptosis in the CD4(high)CD8(high) thymocyte subpopulation. Glycobiology. 2007;17(12):1404–12. Epub 2007/09/26. doi: 10.1093/glycob/cwm104 . 17893094 10.1093/glycob/cwm104

[58] Josefowicz SZ, Lu LF, Rudensky AY. Regulatory T cells: mechanisms of differentiation and function. Annu Rev Immunol. 2012;30:531–64. doi: 10.1146/annurev.immunol.25.022106.141623 . 22224781 10.1146/annurev.immunol.25.022106.141623 PMC6066374

[59] Dominguez-Villar M, Baecher-Allan CM, Hafler DA. Identification of T helper type 1-like, Foxp3+ regulatory T cells in human autoimmune disease. Nature medicine. 2011;17(6):673–5. doi: 10.1038/nm.2389 ; PubMed Central PMCID: PMCPMC3675886. 21540856 10.1038/nm.2389 PMC3675886

[60] Koch MA, Thomas KR, Perdue NR, Smigiel KS, Srivastava S, Campbell DJ. T-bet(+) Treg cells undergo abortive Th1 cell differentiation due to impaired expression of IL-12 receptor beta2. Immunity. 2012;37(3):501–10. doi: 10.1016/j.immuni.2012.05.031 ; PubMed Central PMCID: PMCPMC3501343. 22960221 10.1016/j.immuni.2012.05.031 PMC3501343

[61] Aspelund A, Antila S, Proulx ST, Karlsen TV, Karaman S, Detmar M, et al A dural lymphatic vascular system that drains brain interstitial fluid and macromolecules. J Exp Med. 2015;212(7):991–9. doi: 10.1084/jem.20142290 ; PubMed Central PMCID: PMCPMC4493418. 26077718 10.1084/jem.20142290 PMC4493418

[62] Louveau A, Smirnov I, Keyes TJ, Eccles JD, Rouhani SJ, Peske JD, et al Structural and functional features of central nervous system lymphatic vessels. Nature. 2015;523(7560):337–41. doi: 10.1038/nature14432 ; PubMed Central PMCID: PMCPMC4506234. 26030524 10.1038/nature14432 PMC4506234

[63] Reboldi A, Coisne C, Baumjohann D, Benvenuto F, Bottinelli D, Lira S, et al C-C chemokine receptor 6-regulated entry of TH-17 cells into the CNS through the choroid plexus is required for the initiation of EAE. Nat Immunol. 2009;10(5):514–23. doi: 10.1038/ni.1716 . 19305396 10.1038/ni.1716

[64] Rothhammer V, Heink S, Petermann F, Srivastava R, Claussen MC, Hemmer B, et al Th17 lymphocytes traffic to the central nervous system independently of α4 integrin expression during EAE. The Journal of experimental medicine. 2011;208(12):2465–76. doi: 10.1084/jem.20110434 ; PubMed Central PMCID: PMC3256959. 22025301 10.1084/jem.20110434 PMC3256959

[65] Glatigny S, Duhen R, Oukka M, Bettelli E. Cutting edge: loss of α4 integrin expression differentially affects the homing of Th1 and Th17 cells. Journal of immunology. 2011;187(12):6176–9. doi: 10.4049/jimmunol.1102515 ; PubMed Central PMCID: PMC3237912. 22084440 10.4049/jimmunol.1102515 PMC3237912

[66] Sie C, Korn T, Mitsdoerffer M. Th17 cells in central nervous system autoimmunity. Exp Neurol. 2014;262 Pt A:18–27. doi: 10.1016/j.expneurol.2014.03.009 . 24681001 10.1016/j.expneurol.2014.03.009

[67] Ransohoff RM, Hafler DA, Lucchinetti CF. Multiple sclerosis-a quiet revolution. Nature reviews Neurology. 2015 doi: 10.1038/nrneurol.2015.14 . 25686758 10.1038/nrneurol.2015.14 PMC4556342

[68] Swanton J, Fernando K, Miller D. Early prognosis of multiple sclerosis. Handbook of clinical neurology. 2014;122:371–91. doi: 10.1016/B978-0-444-52001-2.00015-7 . 24507526 10.1016/B978-0-444-52001-2.00015-7

[69] Shen P, Fillatreau S. Antibody-independent functions of B cells: a focus on cytokines. Nat Rev Immunol. 2015;15(7):441–51. doi: 10.1038/nri3857 . 26065586 10.1038/nri3857

[70] Fraussen J, Claes N, de Bock L, Somers V. Targets of the humoral autoimmune response in multiple sclerosis. Autoimmunity reviews. 2014;13(11):1126–37. doi: 10.1016/j.autrev.2014.07.002 . 25108168 10.1016/j.autrev.2014.07.002

[71] Krumbholz M, Derfuss T, Hohlfeld R, Meinl E. B cells and antibodies in multiple sclerosis pathogenesis and therapy. Nature reviews Neurology. 2012;8(11):613–23. doi: 10.1038/nrneurol.2012.203 . 23045237 10.1038/nrneurol.2012.203

[72] Owens GP, Bennett JL, Lassmann H, O'Connor KC, Ritchie AM, Shearer A, et al Antibodies produced by clonally expanded plasma cells in multiple sclerosis cerebrospinal fluid. Annals of neurology. 2009;65(6):639–49. doi: 10.1002/ana.21641 ; PubMed Central PMCID: PMCPMC2843543. 19557869 10.1002/ana.21641 PMC2843543

[73] Mayer MC, Meinl E. Glycoproteins as targets of autoantibodies in CNS inflammation: MOG and more. Therapeutic advances in neurological disorders. 2012;5(3):147–59. doi: 10.1177/1756285611433772 ; PubMed Central PMCID: PMCPMC3349079. 22590479 10.1177/1756285611433772 PMC3349079

[74] Mathey EK, Derfuss T, Storch MK, Williams KR, Hales K, Woolley DR, et al Neurofascin as a novel target for autoantibody-mediated axonal injury. The Journal of experimental medicine. 2007;204(10):2363–72. doi: 10.1084/jem.20071053 ; PubMed Central PMCID: PMCPMC2118456. 17846150 10.1084/jem.20071053 PMC2118456

[75] Derfuss T, Parikh K, Velhin S, Braun M, Mathey E, Krumbholz M, et al Contactin-2/TAG-1-directed autoimmunity is identified in multiple sclerosis patients and mediates gray matter pathology in animals. Proceedings of the National Academy of Sciences of the United States of America. 2009;106(20):8302–7. doi: 10.1073/pnas.0901496106 ; PubMed Central PMCID: PMCPMC2688870. 19416878 10.1073/pnas.0901496106 PMC2688870

[76] Srivastava R, Aslam M, Kalluri SR, Schirmer L, Buck D, Tackenberg B, et al Potassium channel KIR4.1 as an immune target in multiple sclerosis. The New England journal of medicine. 2012;367(2):115–23. doi: 10.1056/NEJMoa1110740 . 22784115 10.1056/NEJMoa1110740 PMC5131800

[77] Brickshawana A, Hinson SR, Romero MF, Lucchinetti CF, Guo Y, Buttmann M, et al Investigation of the KIR4.1 potassium channel as a putative antigen in patients with multiple sclerosis: a comparative study. The Lancet Neurology. 2014;13(8):795–806. doi: 10.1016/S1474-4422(14)70141-3 ; PubMed Central PMCID: PMC4144430. 25008548 10.1016/S1474-4422(14)70141-3 PMC4144430

[78] Villar LM, Sadaba MC, Roldan E, Masjuan J, Gonzalez-Porque P, Villarrubia N, et al Intrathecal synthesis of oligoclonal IgM against myelin lipids predicts an aggressive disease course in MS. The Journal of clinical investigation. 2005;115(1):187–94. doi: 10.1172/JCI22833 ; PubMed Central PMCID: PMCPMC539201. 15630459 10.1172/JCI22833 PMC539201

[79] Villar LM, Masjuan J, Gonzalez-Porque P, Plaza J, Sadaba MC, Roldan E, et al Intrathecal IgM synthesis is a prognostic factor in multiple sclerosis. Annals of neurology. 2003;53(2):222–6. doi: 10.1002/ana.10441 . 12557289 10.1002/ana.10441

[80] Mandrioli J, Sola P, Bedin R, Gambini M, Merelli E. A multifactorial prognostic index in multiple sclerosis. Cerebrospinal fluid IgM oligoclonal bands and clinical features to predict the evolution of the disease. Journal of neurology. 2008;255(7):1023–31. doi: 10.1007/s00415-008-0827-5 . 18535872 10.1007/s00415-008-0827-5

[81] Garcia-Barragan N, Villar LM, Espino M, Sadaba MC, Gonzalez-Porque P, Alvarez-Cermeno JC. Multiple sclerosis patients with anti-lipid oligoclonal IgM show early favourable response to immunomodulatory treatment. Eur J Neurol. 2009;16(3):380–5. doi: 10.1111/j.1468-1331.2008.02504.x . 19175382 10.1111/j.1468-1331.2008.02504.x

[82] Villar LM, Alvarez-Cermeno JC. Comment on the article by Stauch et al. 'Intrathecal IgM synthesis in paediatric MS is not a negative prognostic marker of disease progression: quantitative versus qualitative IgM analysis'. Multiple sclerosis. 2012;18(2):250–1; author reply 2–3. doi: 10.1177/1352458511415890 . 21865414 10.1177/1352458511415890

[83] Ilarregui JM, Croci DO, Bianco GA, Toscano MA, Salatino M, Vermeulen ME, et al Tolerogenic signals delivered by dendritic cells to T cells through a galectin-1-driven immunoregulatory circuit involving interleukin 27 and interleukin 10. Nat Immunol. 2009;10(9):981–91. Epub 2009/08/12. doi: 10.1038/ni.1772 . 19668220 10.1038/ni.1772

[84] Cedeno-Laurent F, Opperman M, Barthel SR, Kuchroo VK, Dimitroff CJ. Galectin-1 triggers an immunoregulatory signature in Th cells functionally defined by IL-10 expression. J Immunol. 2012;188(7):3127–37. doi: 10.4049/jimmunol.1103433 ; PubMed Central PMCID: PMCPMC3311782. 22345665 10.4049/jimmunol.1103433 PMC3311782

[85] Seki M, Oomizu S, Sakata KM, Sakata A, Arikawa T, Watanabe K, et al Galectin-9 suppresses the generation of Th17, promotes the induction of regulatory T cells, and regulates experimental autoimmune arthritis. Clin Immunol. 2008;127(1):78–88. doi: 10.1016/j.clim.2008.01.006 . 18282810 10.1016/j.clim.2008.01.006

[86] Oomizu S, Arikawa T, Niki T, Kadowaki T, Ueno M, Nishi N, et al Galectin-9 suppresses Th17 cell development in an IL-2-dependent but Tim-3-independent manner. Clin Immunol. 2012;143(1):51–8. doi: 10.1016/j.clim.2012.01.004 . 22341088 10.1016/j.clim.2012.01.004

