# Supplementary material for: Galectin-8 as an immunosuppressor in experimental autoimmune encephalomyelitis and a target of human early prognostic antibodies in multiple sclerosis
Source: PLoS One. 2017 Jun 26;12(6):e0177472. doi: 10.1371/journal.pone.0177472 (PMC5484466; doi:10.1371/journal.pone.0177472)
Supplement: S1 Table — Brain sections of the heterocygous knock-in Lgals8+/- mice with positive reaction for β-gal histochemistry are listed as regions considered to express Gal-8. (PDF) [file pone.0177472.s001.pdf]

Supplementary Table I: Gal-8 expression revealed by LacZ histochemistry in the mouse brain.

- Orbital cortex (VO, LO, DLO)
- Primary somatosensory cortex - secondary (S1BF, S1ULp, S2)
- Choroid plexus cells (VL, V3 and V4)
- Part intermediate-lateral septal nucleus (LSI)
- Septo hypothalamic nucleus (SHY)
- Medial preoptic nucleus (MNPO)
- Paraventricular thalamic nucleus (PVA)
- Thalamic nuclei (AD, PVA, AVDM, AVVL, IAD, AM, AMV, VRE, Re, CM)
- Thalamic nuclei (VPL, Rt, VPM, VL, Po, LDVL, LDDM, LPMR, AngT, CL, MHB, LHB, MDC, IMD, CM, PC, PV, fr)
- Lateral thalamic nuclei (VMHVL)
- Dorsomedial thalamic nuclei (VMHDM)
- Ventromedial hypothalamic nucleus (VMHC)
- Oriens Layer hippocampus (Or)
- Core Pregeniculado - magnocellular (PGMC)
- Dorsal lateral geniculate nucleus (DLG)
- Intramedullary thalamic area (IMA)
- Thalamic nuclei (Po, PF)
- Medial vestibular nucleus, parvicellular part (MVePc)
- Dorsomedial periaqueductal gray (DMPAG)
- Ventral zona incerta (ZIV)
- Dorsal Incerta Zone (ZID)
- Periacuaductal Grey Side (LGAP)
- Interpeduncular nucleus (IPL)
- Interpeduncular nucleus, subnucleus intermediate (IPI)
- Interpeduncular nucleus, caudal subnucleus (IPC)
- Red nucleus, magnocellular part (RMC)
- Red nucleus, parvicellular part (RPC)
- Medullary reticular nucleus (MDV)
- Core paranigral the VTA (PN)
- Interpeduncular nucleus, rostral subnucleus (IPR)
- Ventral tegmental nucleus (VTG)
- Pontine reticular nucleus, oral part (PNO)
