# Supplementary material for: Galectin-8 as an immunosuppressor in experimental autoimmune encephalomyelitis and a target of human early prognostic antibodies in multiple sclerosis
Source: PLoS One. 2017 Jun 26;12(6):e0177472. doi: 10.1371/journal.pone.0177472 (PMC5484466; doi:10.1371/journal.pone.0177472)
Supplement: S2 Table — RRMS patients before starting the DMD treatment, with or without Gal-8 autoantibodies in sera, share similar characteristics of age, gender, age at onset, disease duration, EDSS, and presence of baseline gadolinium-enhanced T1 lesions. (PDF) [file pone.0177472.s002.pdf]

Supplementary Table II. Baseline characteristics of RRMS patients with and without anti-Gal-8 autoantibodies

RRMS	Anti-Gal-8 (-)	Anti-Gal-8 (+)	p Value
N°	19	17	
**Gender**<br>**- Female**<br>**- Male**	 12<br>7	 15<br>2	0.13
**Age** (mean years, SD)	32.5 (6.2)	31.1 (8.6)	0.43
**Age at symptoms onset** (mean years, SD)	29.0 (6.7)	27.2 (7.2)	0.49
**Diagnostic delay (mean years, SD)**	1.2 (2.0)	1.4 (2.9)	0.46
**Initial EDSS** (mean, range)	0 (0-2)	0 (0-1.5)	0.93
**Brain MRI**<br>**- GD (+)**<br>**- GD (-)**	 12<br>7	 12<br>5	0.73

RRMS: relapsing remitting multiple sclerosis; SD: Standard deviation; GD: Gadolinium, (+) with, (-) without enhancement. Patients with or without anti-Gal-8 antibodies initially show similar clinical characteristics.
