# Supplementary material for: Galectin-8 as an immunosuppressor in experimental autoimmune encephalomyelitis and a target of human early prognostic antibodies in multiple sclerosis
Source: PLoS One. 2017 Jun 26;12(6):e0177472. doi: 10.1371/journal.pone.0177472 (PMC5484466; doi:10.1371/journal.pone.0177472)
Supplement: S1 File — Daily clinical score of EAE Lgals8+/+ versus Lgals8-/- mice, induced by immunization of an emulsion containing 150 μg of MOG35-55 peptide (MOGp) in 8-12-week-old Lgals8-/- and Lgals8+/+ mice. Daily monitored clinical scale of EAE symptoms: 0, no clinical signs; 1, loss of tail tone; 2, flaccid tail; 3, incomplete paralysis of one or two hind legs; 4, complete hind limb paralysis; 5, moribund 6, death. Data was used to establish daily progression of EAE (Fig 1) and clinical parameters in Table 1. (PDF) [file pone.0177472.s003.pdf]

days	Gal-8 WT																			
1	0	0	0	0	0	0	0	0	0	0	0	0	0	0	0	0	0	0	0	0
2	0	0	0	0	0	0	0	0	0	0	0	0	0	0	0	0	0	0	0	0
3	0	0	0	0	0	0	0	0	0	0	0	0	0	0	0	0	0	0	0	0
4	0	0	0	0	0	0	0	0	0	0	0	0	0	0	0	0	0	0	0	0
5	0	0	0	0	0	0	0	0	0	0	0	0	0	0	0	0	0	0	0	0
6	0	0	0	0	0	0	0	0	0	0	0	0	0	0	0	0	0	0	0	0
7	0	0	0	0	0	0	0	0	0	0	0	0	0	0	0	0	0	0	0	0
8	0	0	0	0	0	0	0	0	0	0	0	0	0	0	0	0	0	0	0	0
9	0	0	0	0	0	0	0	0	0	0	0	0	0	0	0	0	0	0	0	0
10	0	0	0	0	0	0	0	0	0	0	0	0	0	0	1	0	0	0	0	0
11	0	0	0	0	0	0	0	0	2	0	0	0	0	0	2	0	0	2.5	0	0
12	0	0	0	1	2	2	1	0	4	2	0.5	1	0	0	2	0.5	0	2.5	0	0
13	0	0	0	4	2	2	2	0	4	3	1	1	0	0	2		0	2	1	0.5
14	0	0	2	4	2	3	2.5	2	4	4	1	2.5	2	0.5	2	4	0	2	2	1
15	0	0	2	4	2	3	2.5	3	4	4	1.5	2.5	2	3	2.5	4	0	1	4	2
16	0	0	3	4	2	3	2.5	4	4	4	1.5	2.5	3.5	3.5	2	4	0	1.5	4	3
17	0	0	4	4	2	3	1	4	4	4	3	3	4	3.5	2	3	0.5	1.5	3.5	2
18	0	0	4	4	2	2.5	2	4	4	4	3	3	3.5	3.5	2.5	2	1	1	3	2
19	2	0	4	4	2	2	2	4	4	3	4	3.5		3.5	2.5	2	1.5	1	3	1.5
20	2	0	2.5	3	2	3	1	4	4	3.5	4	3		3.5	1.5	1	3	0.5	2	0.5
21	2	0	2	2.5	2	3.5	1	4	4	3.5	4	3	1.5	3.5	1.5	1	3	0.5	2	0.5
22	2	2	2	2.5	2	3	1	4	4	3.5	4	3	1.5	3	1.5	0.5	3.5	0.5	2	0.5
23	2	2	2	2	1	2.5	1	3.5	4	3.5	4	3	1	3	1.5	0.5	4	0.5	1.5	0.5
24	1	3	2	2	1	2.5	1	3.5	4	3.5	4	3	0.5	3	2.5	0.5	4	0.5	1.5	0.5

Gal-8KO																					
0	0	0	0	0	0	0	0	0	0	0	0	0	0	0	0	0	0	0	0	0	0
0	0	0	0	0	0	0	0	0	0	0	0	0	0	0	0	0	0	0	0	0	0
0	0	0	0	0	0	0	0	0	0	0	0	0	0	0	0	0	0	0	0	0	0
0	0	0	0	0	0	0	0	0	0	0	0	0	0	0	0	0	0	0	0	0	0
0	0	0	0	0	0	0	0	0	0	0	0	0	0	0	0	0	0	0	0	0	0
0	0	0	0	0	0	0	0	0	0	0	0	0	0	0	0	0	0	0	0	0	0
0	0	0	0	0	0	0	0	0	0	0	0	0	0	0	0	0	0	0	0	0	0
0	0	0	0	0	0	1	1	0	0	0	0	0	0	0	0	0	0	0	0	0	0
0	0	0	0	0	1	1	4	0	0	0	0	3.5	0	1	0	0	0	0	0	0	0
0	0	0	0	0	1	2	6	0	0	0	0	4	0	4	0	0	0	0	0	0	0
0	0	0	0	0	1	2	6	0	0	0	0	6	0	6	1.5	0	0	0	1.5	0	0
0	0	1	1	0	0	1	6	1	0	0	0	6	0	6	2.5	0	0	0	1.5	1	0
0	0	2	2	1	0	1	6		0	0	0	6	0	6		1	0.5	0	3	2.5	0
0	2	2	4	2.5	0	2	6	2.5	0	0	1.5	6	3	6	3	2	1	0	4	4	0
1	2	2	4	4	0	3	6	4	0	0	3.5	6	3	6	2	3.5	2	1	4	4	0
4	4	2	4	4	0	3	6	3	1	2	3.5	6	3.5	6	1.5	3.5	1.5	2.5	3.5	4	0
4	4	2	4	4	1	0	6	3.5	2.5	4	3	6	3	6	1	4	1.5	2.5	4	4	0.5
4	4	2	3	3.5	2	0.5	6	2.5	3	6	4	6	2.5	6	1	4	3	3.5	3.5	4	1.5
4	4	2	2.5	2.5	6	0	6	2.5	3	6	4	6	2.5	6	1	3.5	2	3	3.5	4	2
4	4	2.5	2.5	2.5	6	0	6	1.5	3	6	2.5	6	2.5	6	1	3.5	2	3	3.5	4	2
4	4	3	4	3	6	0	6	1.5	3	6	2.5	6	2	6	1	3	1.5	2.5	2.5	6	4
4	4	4	4	4	6	0.5	6	1	3	6	2	6	2	6	0.5	3	1	2	2.5	6	4
4	4	4	4	4	6	0	6	0.5	3	6	2	6	1.5	6	0.5	3	1	1.5	2.5	6	4
4	4	4	4	4	6	0	6	2	3	6	1.5	6	1.5	6	0.5	3	1	1.5	2.5	6	4
