# Supplementary material for: Galectin-8 as an immunosuppressor in experimental autoimmune encephalomyelitis and a target of human early prognostic antibodies in multiple sclerosis
Source: PLoS One. 2017 Jun 26;12(6):e0177472. doi: 10.1371/journal.pone.0177472 (PMC5484466; doi:10.1371/journal.pone.0177472)
Supplement: S2 File — Fig 2A: FACS-analyzed frequencies of immune cell subpopulations in splenocytes from 8-12-week-old female Lgals8+/+ (WT) and Lgals8-/- (KO) mice. Table 2A: Frequencies of T cells (CD4+), B cells (CD19+), CD8+ T cells and dendritic cells (CD11c+). Table 2B: CD4+ T cell subpopulations Th1, Th2 and Th17. For polyclonal T cell activation, splenocytes were grown in the presence of 1 μg/ml of αCD3 /μCD28 antibodies for 72 h. For the last 4 h of culture cell were stimulated incubating with 50 ng/ml PMA, 500 ng/ml ionomycin, and 10 μg/ml brefeldin A. Tables show frequencies for Th1, Th2 and Th17 in Lgals8+/+ (WT) and Lgals8-/- (KO) mice in untreated condition (UN) or under polyclonal activation (aCD3/28). Fig 2B: Splenocytes isolated from Lgals8+/+ (WT) and Lgals8-/- (KO) mice analyzed by FACS: (A) Dendritic cells (CD11c+), B cells (CD19+), CD8+ T cells and different CD4+ T cells subsets, naïve (CD44-CD62L+), effector (CD44+CD62L+), memory (CD44+CD62L-) and total cells analyzed in the subset of viable CD4+ CD25- T cells. Results from 4–8 independent experiments show that Gal-8 deficit favors selective Th17 cell differentiation upon polyclonal activation. (PDF) [file pone.0177472.s004.pdf]

Figure 2A

WT	Gal-8 ko		WT	Gal-8 ko		WT	Gal-8 ko		WT	Gal-8 ko
**CD4**			**CD19**			**CD8**			**CD11c**	
14.4	14.1		47	42.1		19	22.3		3.33	4.86
19.8	16.4		57.1	50.9		16	19.9		3.41	3.09
16.2	13.7		40.8	30.6		17.8	23.1		2.58	0.95
14.5	12.5		40.8	61		20.1	16.3		1.52	1.16
16.7	16.1			56			16.1			2.19
				47			13.1			2.42
23	14.8									
17.1	22.1									
16.6	19.8									

Figure 2B

Th1

WT		Gal-8 ko	
UN	aCD3/28	UN	aCD3/28
	4.66		6.33
	4.58		3.56
	10		9.37
0.086	2.07	0.055	1.79
0.027	1.74	0.16	2.96
0.12	2.98	0	0.95
0.008	2.39	0.0045	3.76
0.08	2.18	0.013	1.43

Th17

WT		Gal-8 ko	
UN	aCD3/28	UN	aCD3/28
0.04	1.61	0.041	3.16
0.027	1.94	0.12	4.76
0.16	0.81	0.097	1.69
0.067	1.49	0.18	2.3
0.25	0.92	0.25	1.02

Th2

WT		Gal-8 ko	
UN	aCD3/28	UN	aCD3/28
	1.83		2.15
	0.54		5.62
	3.22		1.69
2.13	4.97	2.61	8.74
2	8.4	2.29	8.86
2.39	4.21	4.87	5.88
2.08	3.81	3.32	5.48
4.07	5.05	4.19	4.79
