# Supplementary material for: Galectin-8 as an immunosuppressor in experimental autoimmune encephalomyelitis and a target of human early prognostic antibodies in multiple sclerosis
Source: PLoS One. 2017 Jun 26;12(6):e0177472. doi: 10.1371/journal.pone.0177472 (PMC5484466; doi:10.1371/journal.pone.0177472)
Supplement: S3 File — (PDF) [file pone.0177472.s005.pdf]

Figure 3
Th1
Frequencies

UN					MOGp			
WT		Gal8$^{-/-}$			WT		Gal8$^{-/-}$	
UN	Gal-8	UN	Gal-8		UN	Gal-8	UN	Gal-8
1.64	0.8	3.19	3.34		3.75	5.63	3.59	3.74
1.6	2.63	2.08	1.68		3.56	3.05	3.67	3.09
1.55	1.39	3.43	2.92		3.81	3.64	5.38	5.42
1.86	1.06	4.43	3.15		4.81	4.05	5.42	0.31
7.79	5.8	5.6	4.37		11.2	5.07	15.9	14.2
7.52	5.23	6.43	5.83		11.3	6.04	12.3	5.48
9.1	4.92	12.7	9.37		7.85	9.05	17.6	12.4
1.21		0.48			3.58		1.72	
1.75		4.36			5.82		4.9	
0.44		1.32			3.44		6.54	
0.33		1.31			1.88		3.8	

Figure 3
Th17
Frequencies

UN				MOGp			
WT		Gal8$^{-/-}$		WT		Gal8$^{-/-}$	
UN	Gal-8	UN	Gal-8	UN	Gal-8	UN	Gal-8
0.25	0.15	0.32	0.11	0.55	1.33	1.49	0.88
0.13	0.21	0.19	0.14	0.58	1.4	1.64	1.16
0.08	0.08	0.13	0.22	0.94	1	1.09	1.26
0.13	0.13	0.22	0.13	1.01	1.15	1.09	1.39
0.37	0.39	0.62	0.43	0.84	0.61	1.29	0.86
0.27	0.21	0.44	0.52	0.71	0.64	1.21	0.73
0.38	0.38	0.72	0.4	1.28	1.14	1.61	1.05
0.07		0.83		1		1.58	
0.19		1.02		2.38		1.34	
0.09		5.78		1.99		2.06	
0.2		0.89		0.93		5.17	
