# Supplementary material for: Galectin-8 as an immunosuppressor in experimental autoimmune encephalomyelitis and a target of human early prognostic antibodies in multiple sclerosis
Source: PLoS One. 2017 Jun 26;12(6):e0177472. doi: 10.1371/journal.pone.0177472 (PMC5484466; doi:10.1371/journal.pone.0177472)
Supplement: S4 File — Splenocytes isolated from Lgals8+/+ (WT) and Lgals8-/- (KO) mice analyzed at steady state for total Tregs (Foxp3+), CXCR3+ and CCR6+ frequency in the Treg (Foxp3+ CD4+) population. Upper Table: Total Tregs obtained from five WT and five Gal8-KO mice (upper table). Middle Table: CXCR3+CCR6-. Bottom Table: CXCR3-CCR6+. Analysis from three WT and three Gal8-KO mice. Data show that galectin-8 deficit increases the frequency of total Tregs and CXCR3+ Tregs. (PDF) [file pone.0177472.s006.pdf]

CD4+FoxP3+

n	WT	KO
**1**	6,14	8,65
**2**	8,92	10,8
**3**	6,71	10,2
**4**	5,02	11,3
**5**	7,12	12,1

CD4+FoxP3
CXCR3+CCR6-

	WT	KO
**1**	0,14	12,8
**2**	0	13,8
**3**	0,93	11,8

CD4+FoxP3+
CXCR3-CCR6+

	WT	KO
**1**	38,3	0,95
**2**	11,1	0,36
**3**	4,39	0
