# Supplementary material for: Galectin-8 as an immunosuppressor in experimental autoimmune encephalomyelitis and a target of human early prognostic antibodies in multiple sclerosis
Source: PLoS One. 2017 Jun 26;12(6):e0177472. doi: 10.1371/journal.pone.0177472 (PMC5484466; doi:10.1371/journal.pone.0177472)
Supplement: S5 File — EAE was induced in wild-type C57BL/6J mice and simultaneously treated (i.p.) with either 100 μg recombinant Gal-8 or PBS (control group) during 20 consecutive days. EAE symptoms monitored daily using the following scale: 0, no clinical signs; 1, loss of tail tone; 2, flaccid tail; 3, incomplete paralysis of one or two hind legs; 4, complete hind limb paralysis; 5, moribund 6, death. (PDF) [file pone.0177472.s007.pdf]

Figure 5A and Table 2

	Vehicle							Gal-8 treatment				
days	1	2	3	4	5	6	7	1	2	3	4	5
0	0	0	0	0	0	0	0	0	0	0	0	0
1	0	0	0	0	0	0	0	0	0	0	0	0
2	0	0	0	0	0	0	0	0	0	0	0	0
3	0	0	0	0	0	0	0	0	0	0	0	0
4	0	0	0	0	0	0	0	0	0	0	0	0
5	0	0	0	0	0	0	0	0	0	0	0	0
6	0	0	0	0	0	0	0	0	0	0	0	0
7	0	0	0	0	0	0	0	0	0	0	0	0
8	0	0	0	0	0	0	0	0	0	0	0	0
9	0	0	0	0	0	0	0	0	0	0	0	0
10	0	0	0	0	0	0	0	0	0	0	0	0
11	1.5	0	0	0	1	1.5	0	0	0	0	0	0
12												
13	2.5	0	3	0	4	3.5	0	1	0	0	0.5	0.5
14	3.5	1.5	2.5	0	4	3.5	1.5	1.5	0.5	0	0	0.5
15	2.5	2	2.5	1	4	3	2	2	2	0.5	0	0
16	2.5	3	3.5	1.5	4	5	2	2	3	2	0	0
17	1.5	3.5	3	2	4	5	3.5	3	3.5	4	0.5	0.5
18	2	4	3.5	3	3	5	4	4	4	3	0.5	0
19												
20	1.5	3.5	4	3.5	2	5	3	2.5	3.5	3.5	1.5	0.5
21	2	4	3	3	2	5	2.5	2.5	4	3.5	2	1
