# Supplementary material for: Galectin-8 as an immunosuppressor in experimental autoimmune encephalomyelitis and a target of human early prognostic antibodies in multiple sclerosis
Source: PLoS One. 2017 Jun 26;12(6):e0177472. doi: 10.1371/journal.pone.0177472 (PMC5484466; doi:10.1371/journal.pone.0177472)
Supplement: S6 File — Number of adherent cells counted in six randomly selected fields after incubation with anti-Gal-8 autoantibodies from three positive (MS10, MS14 and MS20) and three negative patients (MS 21, MS 18 and MS27). Anti-Gal-8 autoantibodies inhibit cell adhesion. (PDF) [file pone.0177472.s008.pdf]

RawData Figure 8A

anti-Gal-8(+)

Patient Sera	MS10	MS14	MS20
	3,0	6,0	7,0
	34,0	3,0	11,0
Number of Adhered Cells	18,0	10,0	15,0
	9,0	5,0	10,0
	5,0	3,0	16,0
	5,0	13,0	7,0

anti-Gal-8(-)

Patient Sera	MS21	MS18	MS27
	18,0	15,0	25,0
	28,0	11,0	27,0
Number of Adhered Cells	27,0	21,0	27,0
	29,0	35,0	23,0
	12,0	18,0	20,0
	23,0	15,0	17,0
