# Supplementary material for: Galectin-8 as an immunosuppressor in experimental autoimmune encephalomyelitis and a target of human early prognostic antibodies in multiple sclerosis
Source: PLoS One. 2017 Jun 26;12(6):e0177472. doi: 10.1371/journal.pone.0177472 (PMC5484466; doi:10.1371/journal.pone.0177472)
Supplement: S7 File — In vitro differentiated Th17 cells from IL-17A-GFP reporter mice were purified based on IL-17A expression (GFP+) and incubated with Gal-8 (20 μg/ml) in the presence of lactose, sucrose or anti-Gal-8 antibodies affinity purified from pooled serum of MS patients. Numbers are the frequency of Annexin V+ 7AAD+ cells of the treated sample relative to the frequency of Annexin V+ 7AAD+ cells of untreated control, representing apoptosis, from three independent experiments. Anti-Gal-8 autoantibodies inhibit Gal-8-induced apoptosis of Th17 cells. (PDF) [file pone.0177472.s009.pdf]

Gate CD4+ Total Apoptosis: Early Apoptosis + Late Apoptosis

	Untreated	Gal8	anti-Gal8	Lactose	Sucrose
**1**	10,7	23,26	12,44	13,53	18,54
**2**	7,97	25,2	13,28	12,63	17,89
**3**	11,77	19,1	14,33	15,37	20,62
