# Supplementary material for: Galectin-8 as an immunosuppressor in experimental autoimmune encephalomyelitis and a target of human early prognostic antibodies in multiple sclerosis
Source: PLoS One. 2017 Jun 26;12(6):e0177472. doi: 10.1371/journal.pone.0177472 (PMC5484466; doi:10.1371/journal.pone.0177472)
Supplement: S8 File — Clinical characteristic of 17 RRMS patients anti-Gal-8(+) and 19 RRMS patients anti-Gal-8(-): N (number of patient); Gender (1 is man and 0 is woman); Age (is age at Anti-Gal-8 assay in years); Age at onset MS (age at first symptoms in years); Diagnostic delay (time between age of onset and age of MS diagnosis); Disease duration (years between onset of MS and age at sample assay); Basal EDSS (EDSS at moment of Anti-Gal assay); Brain MRI Gd+ (number of Gadolinium enhancement T1 lesions at brain at moment of Anti-Gal assay); Spinal MRI Gd+ (number of Gadolinium enhancement T1 lesions at spinal cord at moment of Anti-Gal assay); DMD treatment (DMD treatment after Anti-Gal assay); Final EDSS (EDSS at follow-up); ARR (annual relapses rate in follow-up); Follow-up (years of follow-up after anti-Gal-8 assay). (PDF) [file pone.0177472.s010.pdf]

Anti-Gal-8 positive						
**N**	**gender**	**age**	**age at onset MS**	**diagnostic delay**	**disease duration (years)**	**basal EDSS**
**1**	1	23	20	0	3	1
**2**	0	20	19	0	1	0
**3**	0	42	41	0	1	0
**4**	0	38	35	0	3	0
**5**	0	23	21	0	2	1
**6**	0	30	29	0	1	0
**7**	0	38	36	0	2	1
**8**	1	26	19	5	7	1
**9**	0	38	30	6	8	1,5
**10**	0	33	26	3	7	0
**11**	0	27	23	0	4	0
**12**	0	23	22	0	1	1
**13**	0	22	17	0	5	0
**14**	0	30	29	0	1	0
**15**	0	53	37	10	16	1
**16**	0	32	30	0	2	0
**17**	0	30	29	0	1	0

Anti-Gal-8 negative						
**N**	**gender**	**age**	**age at onset MS**	**diagnostic delay**	**disease duration (years)**	**basal EDSS**
**1**	0	24	22	0	2	1
**2**	0	47	44	0	3	0
**3**	0	43	40	1	3	0
**4**	0	29	26	1	3	0
**5**	1	37	36	0	1	0
**6**	0	27	24	1	3	1
**7**	0	38	30	6	8	0
**8**	1	29	23	0	6	0
**9**	0	28	25	1	3	0
**10**	0	32	30	0	2	1,5
**11**	1	30	27	0	3	0
**12**	1	40	38	0	2	0
**13**	1	37	33	0	4	1
**14**	0	25	19	4	6	1
**15**	1	28	27	0	1	0
**16**	1	31	29	0	2	0
**17**	0	32	26	6	6	0
**18**	0	30	29	0	1	2
**19**	0	31	22	2	9	1

brain MRI Gd+	spinal MRI Gd+	DMD treatment	final EDSS	ARR	Follow-up (years)
1	0	0	1	0,41	0,08
1	0	1	1	0,84	1,33
1	0	1	2	0,42	1,83
1	1	1	0	0,37	1,58
0	0	1	1	0,87	0,75
1	1	2	0	1,74	1,67
0	0	1	1,5	1,14	0,08
1	1	2	1,5	0,80	0,67
1	1	2	4,5	1,01	1,83
0	1	1	1,5	0,29	1,83
0	1	1	1	0,61	1,17
0	0	0	2,5	1,06	0,58
1	0	2	0	0,54	1,67
1	0	0	0	0,55	1,00
1	0	1	5	0,12	0,83
1	1	2	1	1,74	1,08
1	1	1	0	1,11	0,08

brain MRI Gd+	spinal MRI Gd+	DMD treatment	final EDSS	ARR	Follow-up (years)
1	0	2	1	1,01	0,50
1	0	1	0	0,34	2,25
0	0	0	0	0,68	0,08
0	0	1	0	0,39	1,25
1	1	1	1	0,45	1,08
0	0	0	0	0,27	0,25
1	0	0	0	0,25	0,75
1	0	1	1	0,25	0,58
0	0	1	0	0,78	1,42
1	0	1	0	0,89	2,42
1	0	1	1	0,89	2,33
1	0	0	0	0,43	0,08
1	0	2	1	0,84	1,50
1	0	0	1,5	1,92	0,08
1	0	1	0	0,55	0,67
0	0	0	0	0,55	0,58
1	0	0	0	0,68	0,83
0	0	0	0	0,72	0,50
0	0	1	1,5	0,20	2,08
